# The 1,3,5-Triazine Derivatives as Innovative Chemical Family of 5-HT_6_ Serotonin Receptor Agents with Therapeutic Perspectives for Cognitive Impairment

**DOI:** 10.3390/ijms20143420

**Published:** 2019-07-12

**Authors:** Gniewomir Latacz, Annamaria Lubelska, Magdalena Jastrzębska-Więsek, Anna Partyka, Małgorzata Anna Marć, Grzegorz Satała, Daria Wilczyńska, Magdalena Kotańska, Małgorzata Więcek, Katarzyna Kamińska, Anna Wesołowska, Katarzyna Kieć-Kononowicz, Jadwiga Handzlik

**Affiliations:** 1 Department of Technology and Biotechnology of Drugs, Medical College, Jagiellonian University, Medyczna 9, PL 30-688 Cracow, Poland; 2 Department of Clinical Pharmacy, Medical College, Jagiellonian University, Medyczna 9, PL 30-688 Cracow, Poland; 3 Department of Medicinal Chemistry Institute of Pharmacology, Polish Academy of Science, Smętna 12, PL 31-343 Cracow, Poland; 4 Department of Pharmacodynamics, Faculty of Pharmacy, Medical College, Jagiellonian University, Medyczna 9, PL 30-688 Cracow, Poland

**Keywords:** 5-HT_6_ ligands, 1,3,5-triazine, ADME-Tox parameters, pro-cognitive effects, anxiolytic-like activity

## Abstract

Among serotonin receptors, the 5-HT_6_ subtype is the most controversial and the least known in the field of molecular mechanisms. The 5-HT_6_R ligands can be pivotal for innovative treatment of cognitive impairment, but none has reached pharmacological market, predominantly, due to insufficient “druglikeness” properties. Recently, 1,3,5-triazine-piperazine derivatives were identified as a new chemical family of potent 5-HT_6_R ligands. For the most active triazine 5-HT_6_R agents found (**1**–**4**), a wider binding profile and comprehensive in vitro evaluation of their drug-like parameters as well as behavioral studies and an influence on body mass in vivo were investigated within this work. Results indicated the most promising pharmacological/druglikeness profiles for 4-((1H-indol-3-yl)methyl)-6-(4-methylpiperazin-1-yl)-1,3,5-triazin-2-amine (**3**) and 4-((2-isopropyl-5-methylphenoxy)methyl)-6-(4-methylpiperazin-1-yl)-1,3,5-triazin-2-amine (**4**), which displayed a significant procognitive action and specific anxiolytic-like effects in the behavioral tests in vivo together with satisfied pharmaceutical and safety profiles in vitro. The thymol derivative (**4**) seems to be of higher importance as a new lead candidate, due to the innovative, non-indole and non-sulfone structure with the best 5-HT_6_R binding properties.

## 1. Introduction

Among the GPCR serotonin receptors, the 5-HT_6_ subtype (5-HT_6_R) is a most recently identified (1993 in rats, 1996 in human) [1,2] and the most different from the rest due its structure, localization and functions [3]. This receptor has unique structure, including: a short third cytoplasmatic loop and a long C-terminal tail as well as one intron located in the middle of the third cytoplasmatic loop. The 5-HT_6_Rs are almost exclusively located in CNS. Lines of evidence, which described the 5-HT_6_R mRNA found either in human peripheral blood vessels or some animal tissues, have indicated lack of any functional role for the peripheral 5-HT_6_R [3,4,5]. The CNS 5-HT_6_Rs are mainly distributed in the brain areas relevant with cognition, i.e., in the olfactory tubercle, frontal and entorhinal cortices, dorsal hippocampus, nucleus accumbens and striatum [3]. In particular, the localization of 5-HT_6_R in the prefrontal cortex (PFC) is in great importance since PFC is critical to normal cognitive processes, including: Attention, impulsivity, planning, decision-making, working memory, and learning or recall of learned memories [6]. The 5-HT_6_Rs occur in pyramidal cells and GABAergic interneurons of III-V and I layers of PFC, where they regulate neurotransmitter systems involved in the aforementioned cognitive processes. Thus, 5-HT_6_R ligands can be considered as pivotal for innovative treatment of cognitive impairment, with an accent on the 5-HT_6_R antagonists [7]. However, lines of evidence [4,6,7] confirm the similar pharmacological effects for either agonists or antagonists. Both agonists and antagonists have been found to increase cortical performance in different paradigms assessing either cognitive flexibility of learning/memory or to reverse deficits induced by scopolamine and NMDA receptor antagonists (e.g., ketamine and phencyclidine) [8,9,10,11,12]. These results are very promising for therapy but also paradoxical and intriguing from the scientific point of view. Hence, a wider chemical space of 5-HT_6_R ligands is needed to be explored in order to explain this functional paradox.

**Figure 1:**
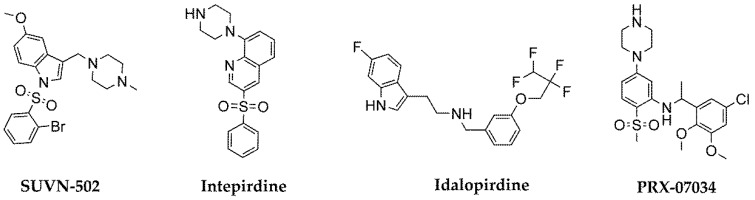
The most advanced 5-HT_6_R agents that have reached clinical trials.

Although a lot of agonists and antagonists have been found for more than 20 years, their structural diversity is poor. The state of art in 2014 indicated that more than 40% of the 5-HT_6_R ligands include indole moieties and more than 80% sulfone ones [12]. This situation has not been significantly changed for the last 4 years [13,14,15,16]. Nevertheless, this huge structurally-similar family has provided a number of compounds with therapeutic perspectives, especially promising for antagonists as potential agents useful in therapy of Alzheimer’s disease. The most advanced compounds: SUVN-502, intepirdine, idalopirdine and PRX-07034 (Figure 1) have reached clinical trials but failed in the latest phases, excluding SUVN-502 that is currently under evaluation in patients with moderate AD on the background of donepezil and memantine (Clinicaltrial.gov identifier: NCT02580305) [17,18,19]. 

Nevertheless, nonselective 5-HT_6_R ligand has been introduced into pharmaceutical market yet. As a main reason of the therapeutic failure, insufficient “drug-likeness” properties of the previously found active 5-HT_6_R agents are mentioned, including: Limited bioavailability, poor blood-brain penetration (brain/plasma < 0.01) and rapid clearance which disqualified compounds in the early stages of drug R&D [20]. Thus, new chemical groups of 5-HT_6_R ligands that exhibit potent receptor affinity and selectivity but also suitable, so called “ADMET profile” (Absorption Distribution Metabolism Elimination Toxicity profile), are strongly desirable to give an alternative for therapy and ensure the success in clinical trials. 

**Figure 2:**
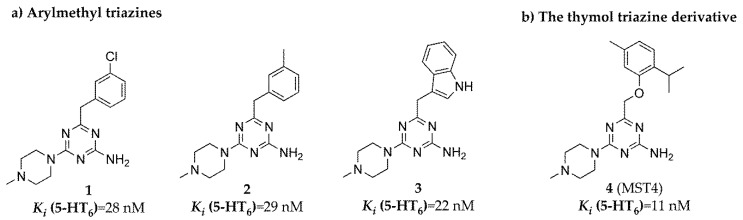
The chemical structures and 5-HT_6_R affinity of 1,3,5-triazine derivatives: (**a**) *m*-chlorophenyl- (**1**), *m*-methylphenyl- (**2**) and 3-indolyl- (**3**) compounds [22,23]; (**b**) 2-isopropyl-5-methylphenoxyl (thymol) derivative (**4**) [24].

In this context, we started to explore the group of 1,3,5-triazine-methylpiperazines as a new chemical space that partly fitted in the pharmacophore features of the 5-HT_6_R antagonists described by Benhamu et al. [21,22,23]. Results of those studies allowed to select a series of derivatives with the high activity for 5-HT_6_R (*K_i_* = 22–29 nM) and satisfying antidepressant-like in vivo effect for arylmethyl derivatives **1–3** (Figure 2) The compounds also displayed an excellent “CNS-drugability” score (4.33–5.58) in Multiparameter Optimization (MPO) assay in silico [22]. Additionally, more potent 5-HT_6_R affinity was found for their thymol analogue MST-4 (**4**, Figure 2) [24], originally synthesized during the search for diuretic agents 45 years ago [25].

Due to the structural originality and promising results in the initial steps of primary pharmacological screening for compounds **1**–**4** [22,23,24], we decided to extend biological studies on them in order to consider an ability of the 1,3,5-triazine 5-HT_6_R ligands as a potential procognitive agents with therapeutic perspectives. Thus, a wider receptor binding profile and the comprehensive in vitro evaluation of their ADMET parameters were explored in this work. Studies in vivo in order to determine potential pro-cognitive effects and anxiolytic-like activity of **l**–**4** as well as an influence on body mass of the most potent 5-HT_6_R agent (**4**) were carried out.

## 2. Results

### 2.1. Radioligand Binding Assay

The 5-HT_6_R ligands **1**–**4** were examined in radioligand binding assays on their affinity and selectivity for the human serotonin receptors: 5-HT_6_, 5-HT_1A_, 5-HT_7b_ and dopaminergic D_2L_ [22,23,24] as well as 5-HT_2_. All receptors were stably expressed in HEK-293 cell line. The results expressed as *K_i_* indicate a very high affinity of compound **4** for 5-HT_6_R (11 nM). Moreover, all examined 1,3,5-triazine-methylpiperazine derivatives showed a significant selectivity over serotoninergic 5-HT_1A_, 5-HT_2A_, 5-HT_7b_ and dopaminergic D_2L_ receptors (Table 1).

### 2.2. ADMET Assays Results

#### 2.2.1. Bioavailability

The penetration through biological membranes is the crucial aspect determining in vivo activity of CNS targeting compounds. Thus, compounds **1**–**4** were tested in the parallel artificial membrane permeability assay (PAMPA) with respect to their ability to passive transport. Moreover, the luminescence Pgp-Glo™ Assay was used in order to determine their influence on P-glycoprotein (Pgp) activity, the main efflux pump in the transport across blood–brain barrier (BBB) and the intestinal epithelium. The assays were performed according to previously described methods and protocols [26,27,28,29]. 

The results of PAMPA were expressed as permeability coefficient *Pe* and shown in comparison to both, the highly permeable (caffeine) and the poorly permeable (norfloxacin) agents (Table 2). 

The examined 5-HT_6_R ligands demonstrated an excellent permeability, with *Pe* values much higher than the breakpoint for permeable compounds (*Pe* ≥ 1.5 × 10^−6^ cm/s). The *Pe* calculated for **1**–**3** were even higher than that estimated for well-permeable caffeine (*Pe* = 15.1 × 10^−6^ cm/s). The highest ability to passive penetration through biological membranes (*Pe* = 23.6 × 10^−6^ cm/s) was shown for the *m*-chlorophenyl derivative (**1**), while the thymol derivative (**4**) was relatively the least permeable.

**Figure 3:**
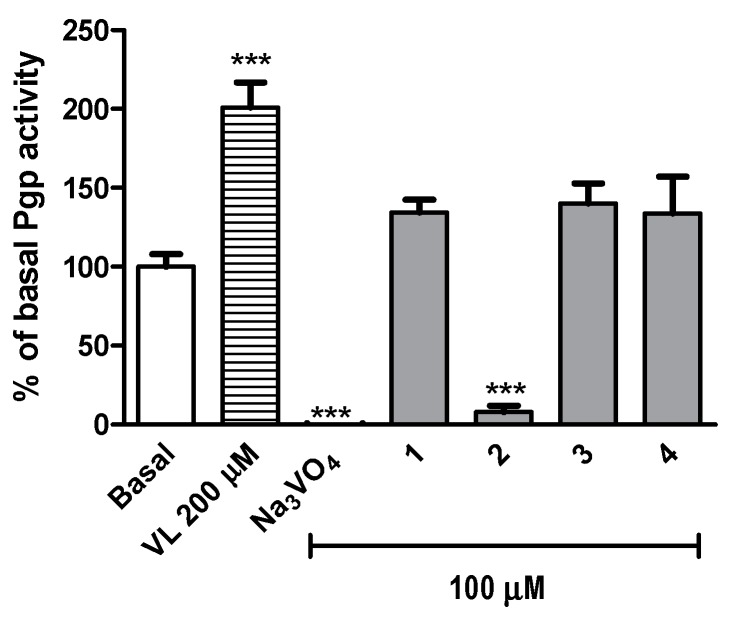
The effect of the Pgp substrate verapamil (VL) (200 μM), Pgp inhibitor Na_3_VO_4_ (100 μM) and compounds **1**–**4** (100 μM) on Pgp basal activity (Basal). The compounds are recognized as a Pgp substrates if they stimulate its basal activity (> 100%), whereas inhibitors reduce Pgp basal activity (< 100%). Data are presented as the mean ± SD. Statistical significance was evaluated by one-way ANOVA, followed by Bonferroni’s comparison test (*** *p* < 0.001 compared with the negative control).

Moreover, compounds **1**, **3** and **4**, tested in the luminescent Pgp-Glo™ Assay System, caused a slight statistically insignificant increase of the basal activity of Pgp up to ~135% (Figure 3). Surprisingly, compound **2**, the *m*-methylphenyl derivative, inhibited significantly (*p* < 0.001) the Pgp basal activity (Figure 3).

#### 2.2.2. Pharmacokinetics

The correlation between compound’s in vitro liver microsomal metabolism and its further faith in vivo allows for understanding the pharmacokinetic properties at the early stage of drug discovery. Thus, the metabolic stability of the best lead candidates (**1**–**4**) was determined by measuring their disappearance with time at 5, 15, 30 and 45 min of incubation with human liver microsomes (HLMs) [26,27]. The *t_1/2_* values and intrinsic clearances (*Cl_int_*) of **1**–**4** were calculated using formulas proposed by Obach [30]. According to the classification bands for HLMs assays [31], all examined compounds (**1**–**4)** were highly stable (*Cl_int_* < 8.6 mL·min^−1^·kg^−1^). The 3-indolyl derivative (**3**) showed the highest metabolic stability with *Cl_int_* = 2.34 mL·min^−1^·kg^−1^ and corresponding *t_1/2_* = 267 min (Table 3).

#### 2.2.3. Metabolic Pathways

**Figure 4:**
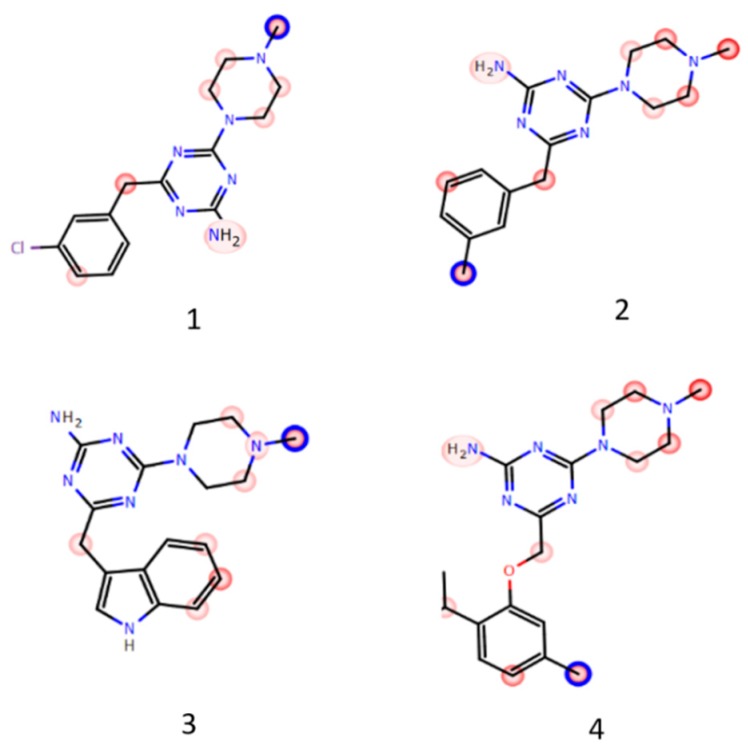
The MetaSite 6.0.1. in silico prediction of the most probable sites of compounds **1**–**4** metabolism. The darker red color, the higher probability to be involved in the metabolism pathway. The blue circle marked the site of compound with the highest probability of metabolic bioconversion.

The prolonged incubation of compounds **1**–**4** with HLMs was applied to determine the metabolic pathways. The obtained in vitro data were supported by in silico metabolism prediction performed by MetaSite 6.0.1 (Figure 4). The software algorithm showed the *N*-methyl substituent at the methylpiperazine moiety as the most probable site of **1** and **3** derivatives metabolic pathway (blue circle marked, Figure 4). The metabolic biotransformations for **2** and **4** were expected mostly at the methyl substituents of *m*-methylphenyl and 2-isopropyl-5-methylphenoxy moieties, respectively (blue circle marked, Figure 4). However, the *N*-methyl substituent of **3** and **4** was also found in silico as a very susceptible site for biotransformation (dark red marked, Figure 4).

As it is shown in Figure 5, compounds **1** and **2** were metabolized by HMLs to give two metabolites (Figure 5A,B), compound **3** three metabolites (Figure 5C), whereas four metabolites were identified for compound **4** (Figure 5D). Subsequently, the MS spectra, MS/MS ion fragmentation analysis and in silico data allowed to determine the most probable metabolic pathways of the all tested 5-HT_6_R ligands (Figures S1–S15, Figure 4, Table 4). The reference reaction was also performed under the same conditions with metabolically unstable drug verapamil (Figure S16).

The in vitro data were in a good accordance with the results obtained in silico, where *N*-methyl substituent was marked as very susceptible for biotransformations (Figure 4). The reaction of demethylation at the *N*-methylpiperazine moiety occurred in all the tested compounds (Figures S3, S6, S9 and S12 in supplementary materials, Table 4). Constantly, the demethylated metabolites were also observed in our previous investigations on 1,3,5-triazine-methylpiperazine derivatives metabolism [32,33]. Additionally, compounds **1**–**4** were found to be metabolised in the reaction of hydroxylation. The MS ion fragment analyses showed that ligands **1**, **3** and **4** were hydroxylated at the piperazine ring (Figures S2, S10 and S14 in supplementary material, Table 4). It is also in accordance with the results obtained in silico, where the carbon atoms of the piperazine ring is marked in red (Figure 4). The hydroxylation site of **2** was determined at the *m*-methylphenyl moiety (Figure S5). Considering in silico data, this hydroxylation occurred the most probably at the methyl substituent (marked in blue, Figure 4). The additional hydroxylation of compound **3** was identified at the 3-indolyl fragment (Figure S8 in supplementary material, Table 4). Two reactions of hydroxylation of compound **4** were determined at the 2-isopropyl-5-methylphenoxy moiety (Figures S13 and S15 in supplementary material). It is very likely, that one of them occurred at the 5-methyl substituent, in a good concordance with the results of in silico simulation (marked in blue, **4**, Figure 4). 

#### 2.2.4. Safety

The comprehensive investigation of safety for compounds **1**–**4** was performed in vitro with the use of recombinant isoforms of cytochrome P450 (CYP), eukaryotic human embryonic kidney HEK-293 and *hepatoma* HepG2 cells as well as the bacterial strain *Salmonella typhimurium* TA100. The growth conditions and assay protocols were applied as described previously [26,27,28,29]. 

**Figure 6:**
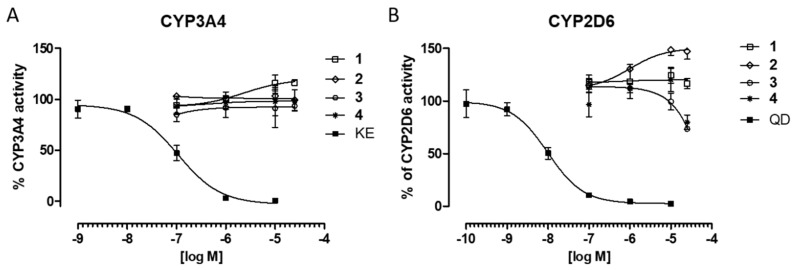
The effect of ketoconazole (KE, IC_50_ = 0.14 µM) and 5-HT_6_R ligands **1**–**4** on CYP3A4 activity (**A**). The effect of quinidine (QD, IC_50_ = 0.01 µM) and 5-HT_6_R ligands **1**–**4** on CYP2D6 activity (**B**).

The potential risk of drug-drug interactions (DDI) were examined by luminescence-based CYP3A4 and CYP2D6 P450-Glo™ assays (Promega^®^). These two CYP isoforms are responsible for metabolism of approximately half of all marketed drugs, and an influence on their activity may be a source of DDI danger [31]. The obtained data showed no influence of **1**–**3** on CYP3A4 activity if comparing to the control reaction. Only a slight induction effect at the highest concentrations of 10 and 25 µM was observed for compound **4** (Figure 6A). The CYP2D6 induction up to ~148% of the control activity was observed in the presence of *m*-methylphenyl derivative **2**, also at concentrations of 10 and 25 µM (Figure 6B). On the other hand, compounds **3** and **4** inhibited CYP2D6 to ~75% of the control activity, but only at their highest concentration of 25 µM (Figure 6B). In conclusion, the slight influence of **1**–**4** on tested CYPs activity in comparison to the respective reference inhibitors: ketoconazole (3A4) and quinidine (2D6), indicates a very low risk of potential DDI.

**Figure 7:**
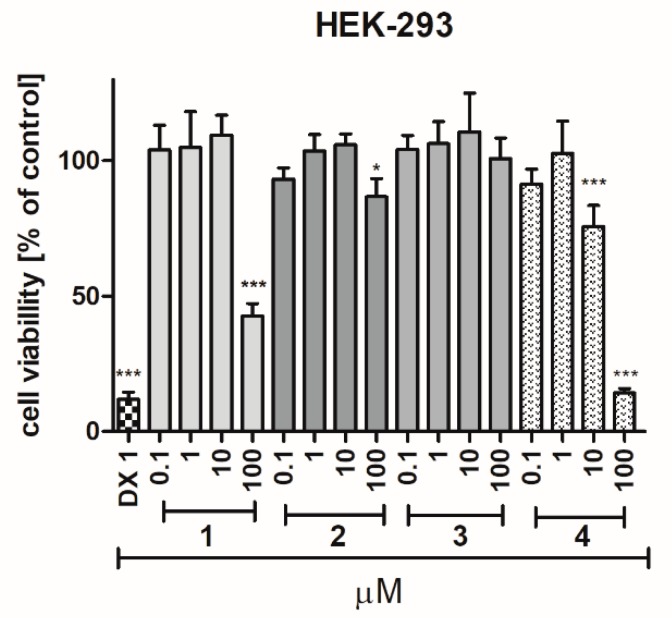
The effects of doxorubicin (DX, 1 µM) and compounds **1**–**4** on human embryonic kidney HEK-293 cell line viability after 72 h of incubation. Statistical significance was evaluated by one-way ANOVA, followed by Bonferroni’s comparison test (* *p* < 0.05, *** *p* < 0.001).

**Figure 8:**
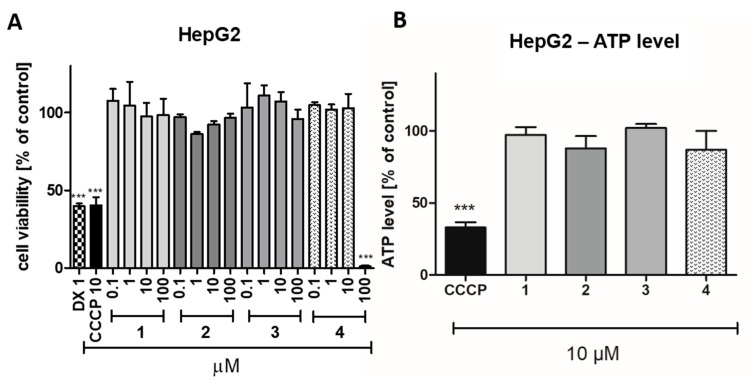
The effects of doxorubicin (DX, 1 µM), mitochondrial toxin carbonyl cyanide 3-chlorophenyl-hydrazone (CCCP, 10 µM) and compounds **1**–**4** on hepatoma HepG2 cell line viability after 72 h of incubation (**A**). The effects of CCCP and compounds **1**–**4** on ATP level in hepatoma HepG2 cell line after 2 h of incubation (**B**). Statistical significance was evaluated by one-way ANOVA, followed by Bonferroni’s comparison test (*** *p* < 0.001).

The cytotoxic effect of compounds **1**–**4** was investigated after 72 h of incubation with HEK-293 and HepG2 cell lines. Among the tested compounds (**1**–**4**), **4** was determined as the most toxic one against HEK-293 cells. The statistically significant decrease in cells viability (*p* < 0.001) was observed at 10 and 100 µM. However, the toxicity of **4** was still much weaker, than toxicity of doxorubicin, used as a positive control (Figure 7). A significant effect was also determined for compound **1** (*p* < 0.001) and compound **2** (*p* < 0.05), but only at the highest used concentration (100 µM). The hepatotoxic effect against HepG2 was observed only for compound **4** at the highest used concentration (100 µM, Figure 8A). In order to complete the obtained data, the bioluminescence based measurement of the ATP level, after a short 2 hour-long exposure of HepG2 cells on compounds **1**–**4** (10 µM), was conducted. As it is shown in Figure 8B, there was no statistically significant decrease of ATP level in hepatoma HepG2 in the presence of the 5-HT_6_ ligands (**1**–**4**), whereas mitochondrial toxin carbonyl cyanide 3-chlorophenylhydrazone (CCCP) decreased the ATP level to ~33% of control. In summary, compounds **1**–**3** appeared to be safe within these assays, whereas a cytotoxic effect for compound **4** was observed at the highest concentration of 100 µM.

**Figure 9:**
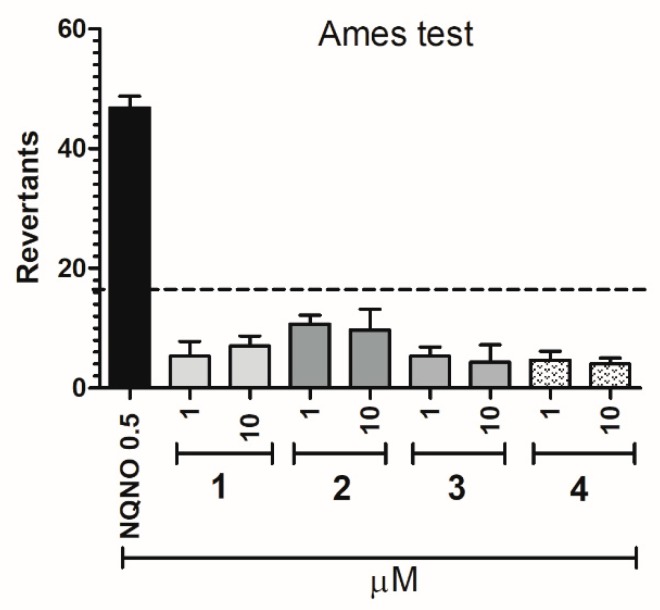
Number of histidine prototrophy revertants of Salmonella typhimurium strain TA100 exposed to the reference mutagen nonyl-4-hydroxyquinoline-N-oxide (NQNO, 0.5 µM) and compounds **1**–**4** in two concentrations 1 and 10 µM. The dashed line marks the mutagen alert.

The potential mutagenicity of **l**–**4** was evaluated by Ames microplate fluctuation protocol (MPF). The used Salmonella typhimurium T100 strain enables the detection of base pair substitution. First, the medium control baseline (MCB) was calculated, which means the number of revertants observed in the control (growth medium + 1% DMSO). According to the manufacturer protocol, the 2-fold increase over the MCB is considered as the mutagen alert. The number of revertants observed in the presence of the examined 5-HT_6_R ligands (**1**–**4**) did not reach this level. Thus, the compounds **1**–**4** did not show any mutagenic effects in vitro (Figure 9).

### 2.3. Behavioral Tests In Vivo

The 1,3,5-triazine derivatives **1**–**4** were investigated in vivo on their anxiolytic-like activity in the Vogel conflict drinking test as well as on their ability to reverse memory impairments using the novel object recognition task (NORT). For compounds displaying the anxiolytic-like action, the hot plate tests were performed in order to estimate if their anxiolytic effect is a specific one. 

#### 2.3.1. Anxiolytic-Like Activity of Investigated Compounds 

**Figure 10:**
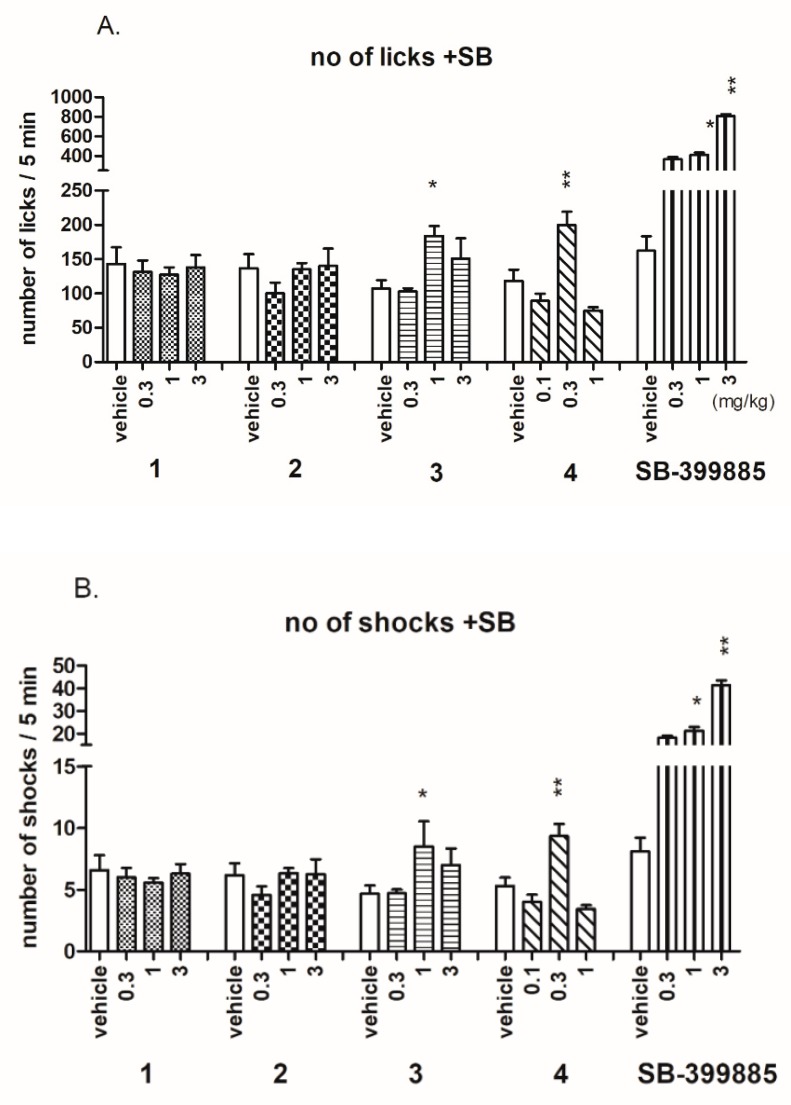
Effects of compounds **1**–**4** and SB-399885 on the number of of licks (**A**) and number of shocks (**B**) accepted in Vogel conflict test in rats. Compounds were administered i.p. 60 min before the test. The animals were observed for 5 min. The data are presented as the mean ± SEM of 6–8 rats. The data were statistically evaluated by one-way ANOVA followed by Bonferroni’s post-hoc test, * *p* < 0.05, ** *p* < 0.01 vs vehicle group.

In the Vogel conflict drinking test, the anxiolytic-like activity was shown only for compounds **3** and **4** and reference 5-HT_6_R antagonist SB-399885 (Figure 10). The compound **4** at the dose of 0.3 mg/kg significantly increased the number of accepted licks (about 70%; ANOVA F(3,23) = 15.653; *p* < 0.0001) as well as the number of licks (about 76%; ANOVA F(3,23) = 15.433; *p* < 0.0001). The compound **3** at the dose of 1 mg/kg significantly increased the number of accepted licks (about 71%; ANOVA F(3,25) = 4.1092; *p* < 0.05) as well as the number of shocks (about 82%; ANOVA F(3,25) = 4.4401; *p* < 0.05). The reference 5-HT_6_R antagonist SB-399885 exerted anxiolytic-like activity in this test at the doses of 1 and 3 mg/kg (Figure 10) [34]. The obtained anxiolytic-like effects in Vogel conflict drinking test seem to be specific since **3** and **4**, administered at doses effective in the Vogel conflict drinking test, had no effect on rat pain reaction time in the hot plate test and did not change amount of water consumed by deprived rats during 5-min session (Table 5).

#### 2.3.2. Ability of Investigated Compounds to Reverse Memory Impairment

**Figure 11:**
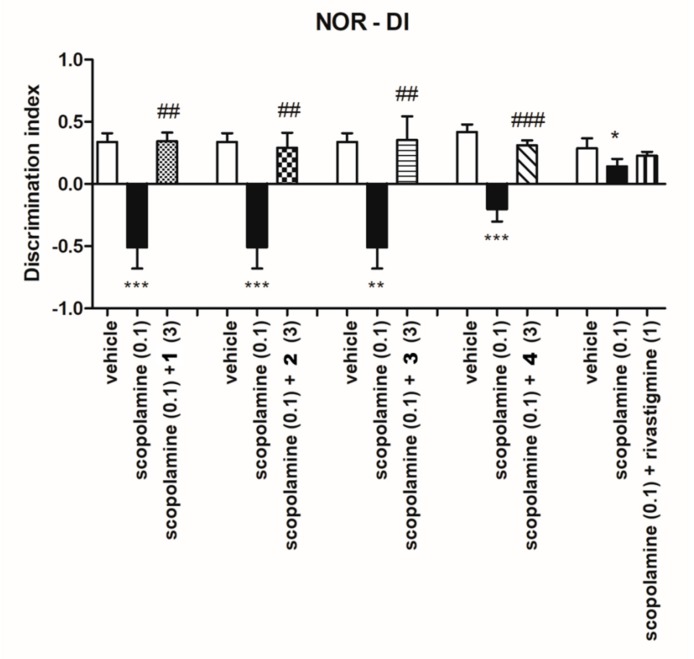
Effects of compounds **1**–**4** on the memory impairment induced by scopolamine in NORT assay. The data are presented as the mean ± SEM of 6–8 rats. The data were statistically evaluated by one-way ANOVA followed by Bonferroni’s post-hoc test, * *p* < 0.05, ** *p* < 0.01 and *p* < 0.001 vs respective vehicle group, and ## *p* < 0.01 and ### *p* < 0.001 vs respective scopolamine treated group. (one-way ANOVA for investigated compounds **1**: F(2,18) = 19.241, *p* < 0.0001; **2**: F(2,18) = 14.154, *p* < 0.001; **3**: F(2,18) = 10.821, *p* < 0.001; **4:** F(2,18) = 21.786; *p* < 0.0001, Rivastigmine: F(2,15) = 3.4051; NS.

Taking into account the ability of various 5-HT_6_R antagonists to improve cognitive deficits [18,35], the NORT assay was used to study the influence of the investigated compounds on visual episodic memory [36]. This test is based on the spontaneous exploration of novel and familiar objects. The administration of scopolamine reduces an ability to discriminate a novel object from a familiar one, and any compounds that have a protective effect against this deficit are regarded as potentially useful for treating cognitive deficits that are characteristic for Alzheimer’s disease and other dementia connected disorders [37,38]. Figure 11 shows that all investigated 5-HT_6_R ligands at the doses of 3 mg/kg significantly ameliorated scopolamine induced learning deficits in the NORT. In the same experimental settings, rivastigmine, at the dose of 1 mg/kg, displayed similar, but lower pro-cognitive effects (Figure 11).

### 2.4. Influence on Body Mass In Vivo

Compound **4**, the most potent 5-HT_6_R agent among investigated group (**1**–**4**), was also evaluated in vivo on its influence on the body mass increase of rats fed with standard or palatable diet. In this context, the control assays of obesity induced with palatable diet and the assays of influence of **4** on body weight, either in model of excessive eating or in that of rats fed with standard diet, were performed.

#### 2.4.1. Obesity Induced with Palatable Diet

**Figure 12:**
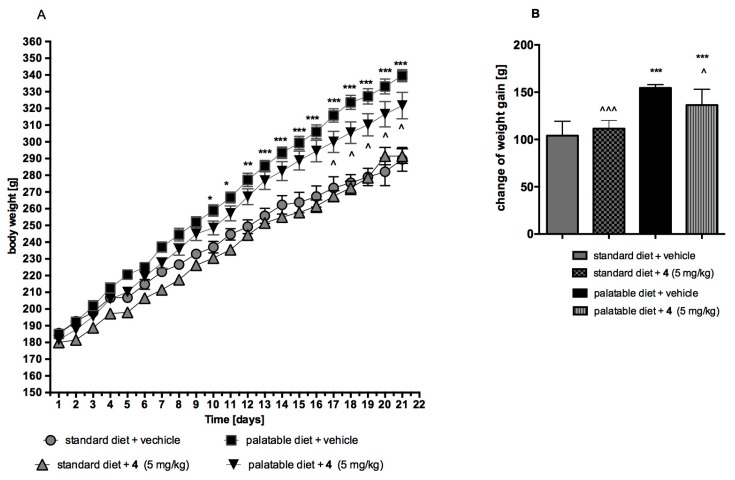
Effect of long-term administration of compound **4** on body weight in male Wistar rats. The change in body weight in Wistar rats fed palatable diet and in Wistar rats fed palatable diet treated for 21 days with the tested compound and the change in body weight in control (standard diet) and in Wistar rats fed standard diet treated for 21 days with the tested compound. Results are means ± SEM, *n* = 6. (**A**) Changes in particular days of experiment. Multiple comparisons were performed by two-way ANOVA, Bonferroni *post-hoc*. * *p* < 0.05, ** *p* < 0.01, *** *p* < 0.001 significant vs. control rats fed standard diet; ^ *p* < 0.05 significant vs. control rats fed palatable diet. (**B**) Changes in whole time of experiment. Multiple comparisons were performed by one-way ANOVA, Dunnett *post-hoc*. * *p* < 0.05, *** *p* < 0.001 significant vs. control rats fed standard diet; ^ *p* < 0.05, ^^^ *p* < 0.001 significant vs. control rats fed palatable diet.

Animals fed with the palatable diet indicated a significantly higher weight gain than the group fed with the standard feed (from day 10 of the experiment). Throughout the experiment, rats from the control group fed with standard feed put on 56.12% of their initial weight, while rats from the control group fed with a palatable feed had a weight gain of 83.56% (Figure 12).

#### 2.4.2. Effect on Body Weight in Model of Excessive Eating and in Rats Fed Standard Diet

The animals fed with palatable feed and treated with **4** showed a significantly less weight gain than animals in the control group consuming the same feed (Figure 12). From day 17 of the experiment, there was a statistically significant difference between the groups. Throughout the experiment, rats from the group fed with palatable feed and treated with **4** put on 71.35% of their initial weight. The body weight of rats treated with **4** and having access to preferential feed differ significantly from the weight of control animals fed standard feed. In contrary, no effects on body weight were noted in animals treated with **4** and consuming standard feed. Throughout the experiment, rats from the group fed with standard feed and treat with **4** put on 61.94% of their initial weight (Figure 12 A,B). 

The similar effects of body weight of rats were observed for previously tested two 1,3,5-triazine derivatives of hydantoin, which also caused the lower body mass increase of the animals fed with fattening feed but did not influence the mass increase caused by standard feeding [39].

## 3. Discussion

According to the assumed goal, the studies have provided a deeper insight into both, pharmacological and “drugability”, profiles of four most active triazine-piperazine 5-HT_6_R agents found within our previous studies. The results obtained allow us to analyze some qualitative structure-activity relationship as the four compounds represent three main structural differences in the field of linker-aromatic fragments, i.e., the 3-substituted benzyl moiety (**1** and **2**), indole-methyl (**3**) and the 2-isopropoxy-5-methylphenoxymethyl one (thymol-methyl, **4**). 

Results of the receptor binding profile indicate the most potent 5-HT_6_R affinity for the thymol derivative **4** as well as a significant selectivity for whole the group above competitive GPCRs (10-763-fold), i.e., 5-HT_1A_, 5-HT_2A_, 5-HT_7_ serotonin and D_2L_-dopamnie receptors (Table 1). However, compounds **1** and **4** displayed the affinity towards 5-HT_2A_R in the submicromolar range (*K_i_* = 272–430 nM) that can play some marginal influence on their pharmacological action in vivo. Hence, the indole derivative **3** demonstrates the best selectivity profile among considered triazines (**1**–**4**), while the thymol derivative **4** is the most potent 5-HT_6_R agent. These results seem to translate into effects in behavioral in vivo studies. Especially, it is seen in the case of the anxiolytic-like activity as only these two compounds (**3** and **4**) displayed statistically significant effect in doses consistent with their activity to the 5-HT_6_ receptors. It is worth to explain that both the compounds caused specific anxiolytic-like effects, confirmed in the hot plate test. The previous studies for some of the compounds indicated also antidepressant-like activity of them in the behavioral assays in rats [22,24,39]. It may suggest an influence of the 1,3,5-triazine derivatives on the monoamine reuptake transporters, which could strengthen their therapeutic potential. However, further experimental data are required to confirm these additional abilities. Nevertheless, the high procognitive activity of whole the series **1**–**4,** demonstrated as an amelioration of scopolamine induced learning deficits, which was much stronger than that of rivastigmine in the NORT, is even more significant and promising result coming from the behavioral studies. Furthermore, the metabolic studies in vivo, performed for the most potent 5-HT_6_R agent **4**, have indicated that this thymol-triazine derivative did not affect the body mass of standardly fed rats, but inhibited the weight gain of fattened animals.

Results of the comprehensive “druglikeness” screening in vitro confirmed the promising properties of the compounds, indicating their very good membrane permeability, low intrinsic clearance and wide pharmaceutical safety, including: a negligible probability of inducing cytotoxic, mutagenic or DDI effects for all tested compounds **1**–**4**. It is worth to accent that a cytotoxic effects against HEK-293 (**1**,**2** and **4**) and HepG2 (**4**) were identified at the compounds concentration much higher than those of doxorubicin and significantly much higher than the active concentration of the compounds in the radioligand binding assays (**4**: *K_i_* = 11 nM vs. the lowest toxic dose 10 μM, i.e ~1000-fold). Thus, all compounds within the series can be considered as pharmaceutically safe.

The global results of the primary screening performed for the 1,3,5-triazine piperazines have confirmed promising properties of this new group in search for novel chemical families of 5-HT_6_R agents with therapeutic perspective as a drug against memory and cognitive impairment, i.e., Alzheimer’s disease and dementia. The qualitative structure-activity relationship analysis indicates the indole- (**3**) and the thymol (**4**) derivatives as two outstanding agents that require wider pharmacological evaluation in the close future. The interesting results obtained for the indole-triazine derivative (**3**) have not been a surprise if considering the role of indole-containing compounds in global search for 5-HT_6_R [12], and only may wonder the highest selectivity of this compound with respect to other 5-HT receptors due to its structural analogies with serotonin. In contrary, our discovery of totally new chemical space represented by the thymol-triazine derivative (**4**) is really innovative and seems to be of great importance in further search for procognitive agents, useful in future therapy of significant civilization diseases. Thus, despite of slightly worse safety and stability profile of **4** if comparing to **3**, this thymol derivative (**4**) seems to be the best candidate for a new lead structure coming from the present studies.

## 4. Materials and Methods 

### 4.1. Radioligand Binding Assay

All used in the radioligand binding assays receptors 5-HT_7b_, 5-HT_6_, 5-HT_1A_, and dopaminergic D_2L_, were stably expressed in HEK-293 cells. Transfections were performed with the use of Lipofectamine 2000. The binding procedure was accomplished via the displacement of 2.5 nM [^3^H]-8-OHDPAT (135.2 Ci/mmol) for 5-HT_1A_R; 2 nM [^3^H]-ketanserin (53.4 Ci/mmol) for 5-HT_2A_R; [3H]-LSD (85.2 Ci/mmol) for 5-HT_6_R; 0.8 nM [^3^H]-5-CT (39.2 Ci/mmol) for 5-HT_7_R; 2.5 nM [^3^H]-raclopride (76.0 Ci/mmol) for D_2L_R. Each compound was tested in triplicate at 7 to 8 different concentrations (10^−11^ to 10^−4^ M). The inhibition constants (*K_i_*) were calculated using the Cheng–Prusoff equation [40] and the results are expressed as the mean of at least two independent experiments.

### 4.2. ADMET

#### 4.2.1. References

The compounds used as the references: caffeine (CFN), carbonyl cyanide 3-chlorophenylhydrazone (CCCP), doxorubicin (DX), ketoconazole (KE), nonyl-4-hydroxyquinoline-*N*-oxide (NQNO), norfloxacin (NFX) and quinidine (QD) were obtained from Sigma-Aldrich (St. Louis, MO, USA). The references for Pgp activity studies: verapamil (VL) and Na_3_VO_4_ were provided with the luminescent Pgp-Glo™ Assay System (Promega, Madison, WI, USA).

#### 4.2.2. Bioavailability

The used 96-wells Pre-coated PAMPA Plate System Gentest™ was obtained from Corning, (Tewksbury, MA, USA). The tested 5-HT_6_R ligands and the references (CFN and NFX) solutions (all at 200 µM) were prepared in PBS buffer (pH = 7.4) and added to the donor wells (300 μL/well). 200 μL/well of PBS was added to the acceptor wells. All compounds were analyzed in triplicate. The plate was incubated at room temperature for 5 h. Then, the 50 μL was aspirated from each well and diluted next with 50 µL solution of an internal standard (IS). The compounds’ concentrations in acceptor and donor wells were estimated by the UPLC-MS analyses, which were performed by LC/MS Waters ACQUITY™ TQD system with the TQ Detector (Waters, Milford, USA). The permeability coefficients (*Pe*, cm/s) were calculated according described previously formulas [26,27,28,29,41]. According to the PAMPA plate’s manufacturer compounds with *Pe* values higher than 1.5 × 10^−6^ cm/s possess good human oral absorption capacity [41].

The luminescent Pgp-Glo™ Assay System used for determination of 5HT_6_R ligands’ influence on P-glycoprotein activity was purchased from Promega (Madison, WI, USA). The assay was performed in triplicate as described previously [26,27,28]. Compounds **1**–**4** (100 μM) were incubated with Pgp membranes for 40 min at 37 °C. The references: VL and Na_3_VO_4_ were incubated at 200 and 100 µM, respectively. The luminescence signal was measured by microplate reader EnSpire PerkinElmer (Waltham, MA, USA).

#### 4.2.3. Pharmacokinetics

The pharmacokinetic parameters of compounds **1**–**4** were estimated by using human liver microsomes (HLMs) (Sigma-Aldrich, St. Louis, MO, USA), according to the described previously protocols [26,27]. The disappearance of 5-HT_6_R ligands (50 µM) in the presence of HLMs (1 mg/mL) was determined at 5, 15, 30 and 45 min of incubation in 10 mM Tris–HCl buffer (37 °C) by LC/MS Waters ACQUITY™ TQD system with the TQ Detector (Waters, Milford, USA). The *t_1/2_* values and intrinsic clearances (*Cl_int_*) were calculated using IS by protocols and formulas proposed by Obach [30]. 

The in vitro evaluation of metabolic pathways was performed by prolonged, 120 min incubation of compounds **1**–**4** with HLMs. The concentration of tested 5-HT_6_R ligands as well as the ingredients of the reaction mixture were similar to those described above pharmacokinetics assays. The LC/MS analyses with additional MS ion fragmentation of the products and substrates were performed to determine the most probable structures of 5-HT_6_R ligands’ metabolites.

The in silico prediction of metabolic biotransformations was performed by MetaSite 6.0.1 software (Molecular Discovery Ltd, Hertfordshire, UK) [42]. The computational liver model of metabolism was used for determination of the most probable sites of metabolism and identification of structures of obtained in vitro metabolites.

#### 4.2.4. Safety

The luminescent CYP3A4 P450-Glo™ and CYP2D6 P450-Glo™ Promega^®^ (Madison, WI, USA) were used for investigation of potential drug-drug interactions. All assays and protocols were described before [26,27,28]. The compounds were tested in triplicate at the final concentrations in range from 0.01 to 25 μM. The luminescent signal was measured by using a microplate reader EnSpire PerkinElmer (Waltham, MA, USA).

The cytotoxicity of compounds **1**–**4** was evaluated with use of human embryonic kidney HEK-293 (ATCC^®^ CRL-1573™) and *hepatoma* HepG2 (ATCC^®^ HB-8065™). Cells were grown under described previously conditions [26,27,28,29]. Compounds **1**–**4** were incubated at 96-wells plate with cells for 72 h in the final concentration range (0.1–100 μM), whereas the references CCCP and DX at 10 μM and 1 μM, respectively. The cells’ viability was determined by CellTiter 96^®^ AQueous Non-Radioactive Cell Proliferation Assay (MTS), which was purchased from Promega (Madison, WI, USA). The absorbance was measured using a microplate reader EnSpire (PerkinElmer, Waltham, MA USA) at 490 nm. All compounds were tested in quadruplicate. For ATP-level measurement the cells were growth in similar way as in cytotoxicity assays. The CellTiter-Glo^®^ Luminescent Cell Viability Assay and protocol was provided by Promega (Madison, WI, USA). The luminescence was measured after 2 hours-long cells’ exposition on tested compounds and the positive control CCCP by a microplate reader EnSpire (PerkinElmer, Waltham, MA USA) at luminescence mode. All compounds were tested in triplicate.

The mutagenicity of 5-HT_6_R ligands was evaluated by Ames microplate fluctuation protocol (MPF) obtained from Xenometrix, (Allschwil, Switzerland). The used *Salmonella typhimurium* TA100 strain has base pair substitution (hisG46 mutation, which target is GGG). The experiments were performed as described before [26,27,28]. The compounds **1**–**4** were tested in two final concentrations, 1 and 10 μM, in triplicate. The occurrence of revertants was visualized by pH indicator dye which was present in the bacterial medium. The color changes from violet to yellow were confirmed by measurements of absorbance with a microplate reader (EnSpire) at 420 nm.

### 4.3. Behavioral Tests In Vivo

#### 4.3.1. Animals

The experiments were performed on male Wistar rats (200–250 g) obtained from an accredited animal facility at the Jagiellonian University Medical College, Poland. The animals were housed in group of four in controlled environment (ambient temperature 21 ± 2 °C; relative humidity 50–60%; 12-h light/dark cycles (lights on at 8:00). Standard laboratory food (LSM-B) and filtered water were freely available. Animals were assigned randomly to treatment groups. All the experiments were performed by two observers unaware of the treatment applied between 9:00 and 14:00 on separate groups of animals. All animals were used only once. Procedures involving animals and their care were conducted in accordance with current European Community and Polish legislation on animal experimentation. Additionally, all efforts were made to minimize animal suffering and to use only the number of animals necessary to produce reliable scientific data. 

The experimental protocols and procedures described in this manuscript were approved by the I Local Ethics Commission in Cracow (no 293/2015) and complied with the European Communities Council Directive of 24 November 1986 (86/609/EEC) and were in accordance with the 1996 NIH Guide for the Care and Use of Laboratory Animals. 

#### 4.3.2. Drugs

The following drugs were used: scopolamine (hydrobromide, Tocris, Uk), rivastigmine (tatrate, Tocris, Uk) and SB-399885 (N-[3,5-dichloro-2-(methoxy)-phenyl]-4-(methoxy)-1-(piperazinyl)benzenesulfonamide; GlaxoSmithKline, UK). All compounds were suspended in 1% Tween 80 immediately before administration in a volume of 2 mL/kg. Investigated compounds were administered intraperitoneally (i.p.) 60, while scopolamine was given subcutaneously (s.c.) and SB-399885 i.p. 30 min before testing. Control animals received vehicle (1% Tween 80) according to the same schedule. 

#### 4.3.3. Vogel Conflict Drinking Test

The testing procedure was based on a method of Vogel et al. (1971) [43] and used Anxiety Monitoring System “Vogel test” produced by TSE Systems (Bad Homburg, Germany). It was consisted of polycarbonate cages (dimensions 26.5 cm × 15 cm × 42 cm), equipped with a grid floor made from stainless steel bars and drinking bottles containing tap water. Experimental chambers were connected to PC software by control chassis and electric shocks’ generator. On the first day of the experiment, the rats were adapted to the test chambers and drink water from the bottle spout for 10 min. Afterwards, the rats were returned to their home cages and were given 30 min free access to water followed by a 24-h water deprivation period. The adaptation session and water deprivation protocols were repeated on the second day of the experiment. On the third day the rats were placed again in the test chambers 60 min after investigated and reference compounds administration and were given free access to the drinking tube. Recording data started immediately after the first lick and rats were punished with an electric shock (0.5 mA, lasting 1 s) delivered to the metal drinking tube every 20 licks. The number of licks and the number of shocks received during a 5-min experimental session were recorded automatically.

#### 4.3.4. Hot Plate and Free-Drinking Tests 

To control possible drug-induced changes in shock sensitivity or the thirst drive (which may contribute to animals’ behavior in the Vogel conflict drinking test), stimulus threshold and water consumption during a free-drinking session were determined in separate groups of rats. In either of those two studies, the rats were manipulated similarly to the Vogel conflict drinking test, including two 24-h water deprivation periods separated by 10 min adaptation session in experimental cages and 30 min of water availability in their home cages. In the free-drinking test, each animal was allowed to freely drink from the waterspout and the amount of water (g) consumed during 5 min was recorded for each rat. 

The pain threshold was investigated using hot plate test (Commat Ltd, Ankara, Turkey) in rats [44]. The plate was enclosed with a transparent Plexiglass cylinder (35 cm high) to keep the animal on the heated surface of the plate. The latency to pain reaction (licking a hind paw or jumping) when the rat was placed on a hot plate (52.5 ± 0.5 °C, 19 cm diameter) was measured. The rat was removed from the plate immediately upon visible pain reaction or if no response occurred within 30 s.

#### 4.3.5. Novel Object Recognition Test (NORT)

Five days before the experiment, the rats were transferred to the laboratory, labeled and, thereafter, left to acclimate to the new environment. The animals were handling every five days before experiments to minimize the stress reaction. The protocol was adapted from the original work [37,45]. The test session comprising of two trials separated by an inter-trial interval (ITI) of 1 h was carried out on the next day. During the first trial (familiarization, T1) two identical objects (A1 and A2) were presented in the opposite corners of the open field, approximately 10 cm from the walls. During the second trial (recognition, T2) one of the A objects was replaced by a novel object B, so that the animals were presented with the A = familiar and B = novel objects. Both trials lasted for 3 min and the animals were returned to their home cages after T1. The objects used were the metal Coca-Cola cans and the glass jars filled with the sand. The heights of the objects were comparable (~12 cm) and the objects were heavy enough not to be displaced by the animals. The sequence of presentations and the location of the objects were randomly assigned to each rat. After each measurement, the floor was cleaned and dried.

The animals explored the objects by looking, licking, sniffing or touching the object but not when leaning against, standing or sitting on the object. Any rat exploring the two objects for less than 5 s within 3 min of T1 or T2 was eliminated from the study. Exploration time of the objects was measured by blind experimenter. Based on exploration time (E) of two objects during T2, discrimination index (DI) was calculated according to the formula: DI = (EB − EA)/(EA + AB). Using this metric, scores approaching zero reflects no preference while positive values reflect preference for the novel object and negative numbers reflect preference for the familiar.

Scopolamine, used to attenuate learning, was administered at the dose of 0,1 mg/kg (s.c.) 30 min before familiarization phase (T1), while investigated compounds were given 60 min before T1 session.

### 4.4. Assays of Influence on Body Mass In Vivo

#### 4.4.1. Animals

The experiments were carried out on male Wistar rats. Initial body weight was: 170–195 g. The animals were housed in pairs in plastic cages in constant temperature facilities exposed to a light-dark cycle; water and food were available ad libitum. Control and experimental groups consisted of six animals each. The animals were housed in controlled environment (ambient temperature 21 ± 2 °C; relative humidity 50%–60%; 12-h light/dark cycles (lights on at 5:00). Standard laboratory food (LSM-B) and filtered water were freely available. Animals were assigned randomly to treatment groups. All the experiments were performed by two observers unaware of the treatment applied between 9:00 and 14:00 on separate groups of animals. All animals were used only once. 

The experimental protocols and procedures described in this manuscript were approved by the I Local Ethics Commission in Cracow (no 293/2015) and complied with the European Communities Council Directive of 24 November 1986 (86/609/EEC) and were in accordance with the 1996 NIH Guide for the Care and Use of Laboratory Animals. 

#### 4.4.2. The Effect of 4 on Body Weight of Non-Obese Rats Fed Palatable Diet (Model of Excessive Eating) 

The influence of **4** on body weight in the model of excessive eating was assessed [46,47]. Male Wistar rats (170–190 g) were housed in pair. Two groups of 6 rats were fed diets consisting of milk chocolate with nuts, cheese, salted peanuts, and 7% condensed milk and also had access to standard feed (Labofeed B, Morawski Manufacturer Feed, Poland) and water ad libitum for 3 weeks. Palatable control group (palatable diet + vehicle) received vehicle (1% Tween 80, intraperitoneally), while palatable test group (palatable diet + **4**) was injected (intraperitoneally) with **4** at the dose 5 mg/kg b.w. dissolved in 1% Tween 80. Body weights were measured daily immediately prior to administration of drugs. 

Palatable diet contained: 100 g peanuts—614 kcal; 100 ml condensed milk—131 kcal; 100 g milk chocolate with hazelnuts—529 kcal; 100 g cheese (Greek type)—270 kcal. Standard diet contained 100 g feed—280 kcal.

#### 4.4.3. The Effect of 4 on Body Weight of Non-Obese Rats Fed Only with Standard Diet

Male Wistar rats (170–190 g) were housed in pair. Control group (standard diet + vehicle) received vehicle (1% Tween 80, intraperitoneally), while the test group (standard diet + **4**) was injected (intraperitoneally) with **4** at the dose 5 mg/kg b.w. dissolved in 1% Tween 80. Body weights were measured daily immediately prior to administration of drugs. 

### 4.5. Statistical Analysis

The GraphPad Prism™ 6 software (GraphPad Software, La Jolla, CA, USA) was used to calculate statistical significances and IC_50_ values in ADMET and in vivo experiments. 

The statistical significances were evaluated by an analysis of variance one-way ANOVA followed by Bonferroni’s post hoc test (statistical significance set at *p* < 0.05) in ADMET, anxiolytic-like, and NOR test. 

Statistical significance in influence on body mass was calculated using one-way ANOVA with Dunnett’s multiple comparison test post-hoc or two-way ANOVA with Bonferroni multiple comparison test post-hoc. Differences were considered statistically significant at: *,^ *p* ≤ 0.05, **,^^ *p* ≤ 0.01, ***,^^^ *p* ≤ 0.001. Results are given as arithmetic means with a standard error of the mean.

## 5. Conclusions

The current world-research on serotonin receptors indicates great abilities of 5-HT_6_ receptor ligands for the potential therapy of the topic civilization CNS diseases, with an accent on cognitive impairments. Consequently, too little chemical diversity of the previously found 5-HT_6_ agents strongly emphasizes the need to explore new chemical spaces. In this context, our studies allowed to find four piperazine derivatives of 1,3,5-triazine that displayed high selectivity and strong nanomolar affinity for the 5-HT_6_R as well as CNS-activity and beneficial ADMET profile in primary screening in vitro and in vivo, respectively. Results of this work indicate the most promising pharmacological and “druglike” profile for two compounds, 4-((1H-indol-3-yl)methyl)-6-(4-methylpiperazin-1-yl)-1,3,5-triazin-2-amine (**3**) and 4-((2-isopropyl-5-methylphenoxy)methyl)-6-(4-methylpiperazin-1-yl)-1,3,5-triazin-2-amine (**4**), which have displayed both the potent procognitive action and specific anxiolytic-like effects in the behavioral tests in vivo as well as very good (**3**) or good (**4**) pharmaceutical- and safety profiles in vitro. Although the indole derivative (**3**) was predominant in terms of either 5-HT_6_R selectivity or ADMET-profile, the thymol derivative (**4**) seems to be of higher importance due to the innovative non-indole and non-sulfone structure and the best 5-HT_6_R binding properties. Thus, the compound **4** can be selected as a new lead structure for further modifications in the search of 5-HT_6_R agents with therapeutic perspectives, while both **3** and **4** may be useful for next stages either in the search for procognitive drug candidate or as tool-compounds for various studies on molecular mechanisms involving the 5-HT_6_ receptors.

## Figures and Tables

**Figure 5:**
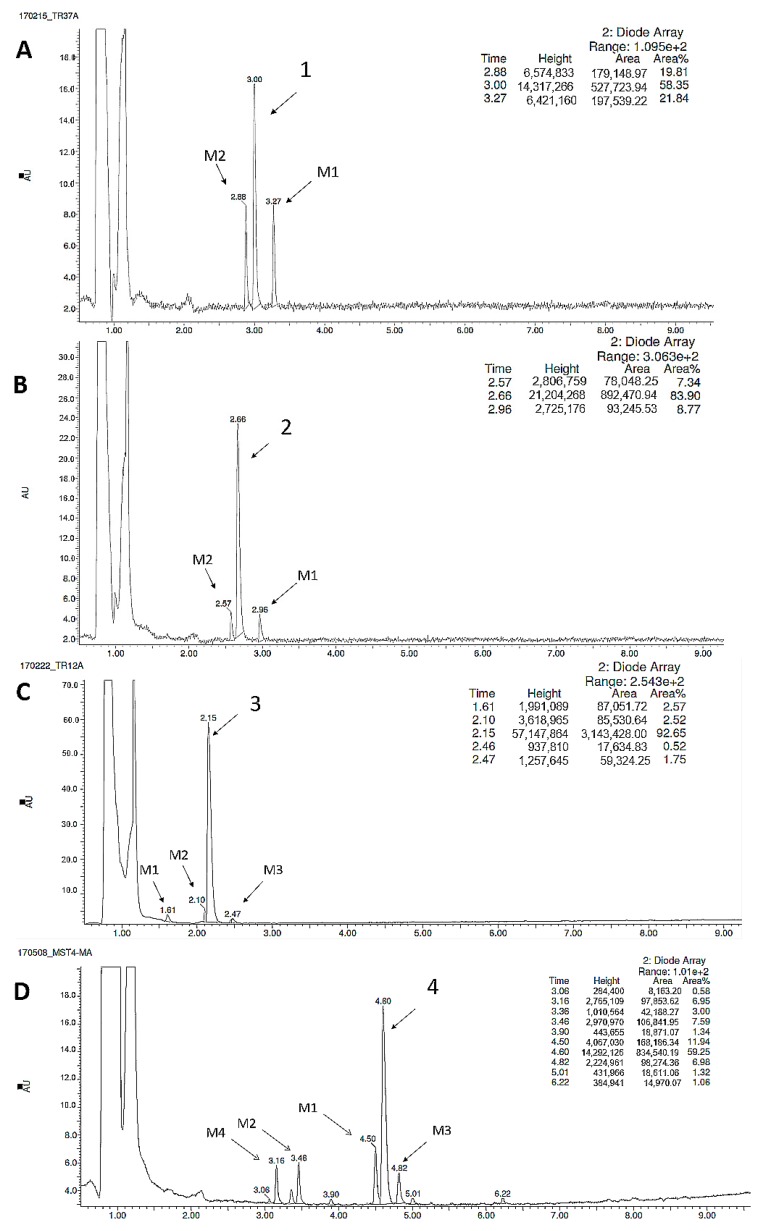
UPLC spectra of the reaction mixtures after 120 min incubation of compounds **1** (**A**), **2** (**B**), **3** (**C**), and **4** (**D**) with HLMs.

**Table 1:** The affinities for serotonin/dopamine receptors of the compounds **1**–**4**.

Cpd	*K_i_* (nM)				
	5-HT_6_ [^3^H]-LSD	D_2L_ [^3^H]- Raclopride	5-HT_1A_ [^3^H]-8-OH-DPAT	5-HT_2A_ [^3^H]- Ketanserin	5-HT_7_ [^3^H]-5-CT
**1**	28 ^a^	838 ^a^	4954 ^a^	272	8710 ^a^
**2**	29 ^a^	1192 ^a^	10,220 ^a^	329	16,160 ^a^
**3**	22 ^a^	1946 ^a^	9453 ^a^	1757	16,790 ^a^
**4**	11 ^a^	1094	12,530	430	11,950

^a^ Receptors affinity that was determined previously [22,23,24].

**Table 2:** The results obtained in PAMPA for 5-HT_6_R ligands and the references.

Comp.	*^1^Pe* (10^−6^ cm/s) ± SD
**CFN**	15.1 ± 0.4
**NFX**	0.56 ± 0.1
**1**	23.6 ± 2.14
**2**	19.9 ± 2.68
**3**	17.9 ± 5.76
**4**	12.3 ± 1.98

^1^ PAMPA plate’s manufacturer breakpoint for permeable compounds: *Pe* ≥ 1.5 × 10^−6^ cm/s.

**Table 3:** The pharmacokinetic properties of compounds **1**–**4**.

Comp.	*t_1/2_* (min)	*^1^Cl_int_* (mL·min^−1^·kg^−1^)
**1**	83	7.56
**2**	158	3.96
**3**	267	2.34
**4**	99	6.30

^1^ The classification bands for HLMs assays: *Cl_int_* ≤ 8.6 mL for low clearance compounds [30].

**Table 4:** The molecular masses and metabolic pathways of compounds **1**–**4**.

Substrate	Molecular Mass (*m/z*)	Amount of Metabolites	Molecular Mass of the Metabolite (*m/z*)	Metabolic Pathway
**1**	319.13	2	335.08 (**M1**)	*hydroxylation*
			305.11 (**M2**)	*demethylation*
**2**	299.19	2	315.14 (**M1**)	*hydroxylation*
			285.10 (**M2**)	*demethylation*
**3**	324.18	3	340.33 (**M1**)	*hydroxylation*
			310.18 (**M2**)	*demethylation*
			340.13 (**M3**)	*hydroxylation*
**4**	357.21	4	342.19 (**M1**)	*demethylation*
			373.23 (**M2**)	*hydroxylation*
			373.23 (**M3**)	*hydroxylation*
			373.23 (**M4**)	*hydroxylation*

**Table 5:** Effect of compound **3** and **4** in the hot plate and water consumption tests in water-deprived rats.

Treatment	Dose (mg/kg)	Hot Plate Test Time of Reaction (s)	Water Consumption (g/5 min)
**Vehicle**	0	12.4 ± 1.4	4.3 ± 0.4
**3**	1	10.8 ± 1.2	4.4 ± 0.1
		F(1,12) = 0.6934; ns	F(1,14) = 0.0635; ns
**4**	0.3	13.8 ± 1.5	4.6 ± 0.2
		F(1,13) = 0.4406; ns	F(1,14) = 0.2834; ns

Compounds were injected *i.p.* 60 min before the test. Values represent the means ± SEM of time reaction in the hot plate test and amount of water consumed during 5-min test session. The data were analyzed using one-way ANOVA, followed by Bonferroni‘s post hoc test; ns = non-significant. *N* = 7–8.

## Supplementary Materials

Supplementary materials can be found at https://www.mdpi.com/1422-0067/20/14/3420/s1.
