# Supplementary material for: The 1,3,5-Triazine Derivatives as Innovative Chemical Family of 5-HT6 Serotonin Receptor Agents with Therapeutic Perspectives for Cognitive Impairment
Source: Int J Mol Sci. 2019 Jul 12;20(14):3420. doi: 10.3390/ijms20143420 (PMC6678253; doi:10.3390/ijms20143420)
Supplement: Supplementary file 1 [file ijms-20-03420-s001.pdf]

Supplementary material

The 1,3,5-triazine derivatives as innovative chemical family of 5-HT$_6$ serotonin receptor agents with therapeutic perspectives for cognitive impairment

Gniewomir Latacz, Annamaria Lubelska, Magdalena Jastrzębska-Więsek, Anna Partyka, Małgorzata Anna Marć, Grzegorz Satała, Daria Wilczyńska, Magdalena Kotańska, Małgorzata Więcek, Katarzyna Kamińska, Anna Wesołowska, Katarzyna Kieć-Kononowicz, Jadwiga Handzlik

Table of contents:

1. MS spectra and MS ion fragment analyses of compound **1** and its metabolites 1
2. MS spectra and MS ion fragment analyses of compound **2** and its metabolites 3
3. MS spectra and MS ion fragment analyses of compound **3** and its metabolites 4
4. MS spectra and MS ion fragment analyses of compound **4** and its metabolites 6
5. UPLC spectra of the reference reaction with HLMs……………………………….8

1. MS spectra and MS ion fragment analyses of compound 1 and its metabolites.

A

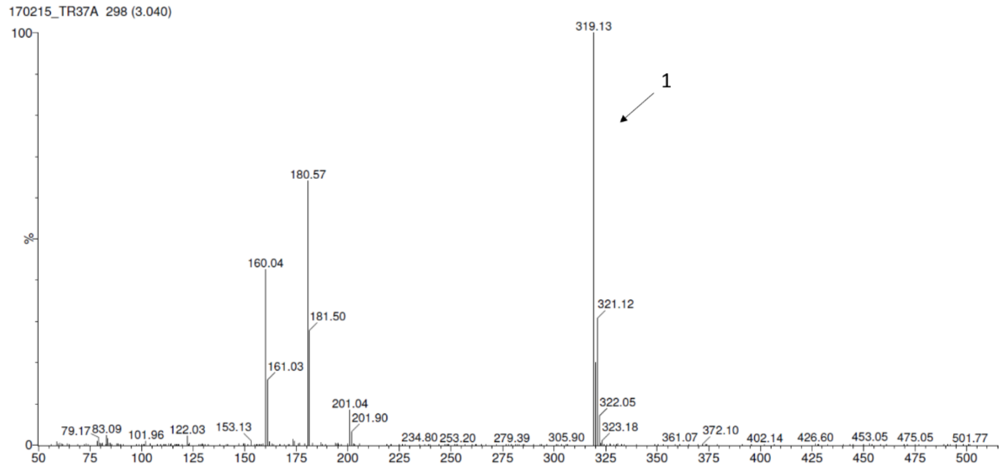

B

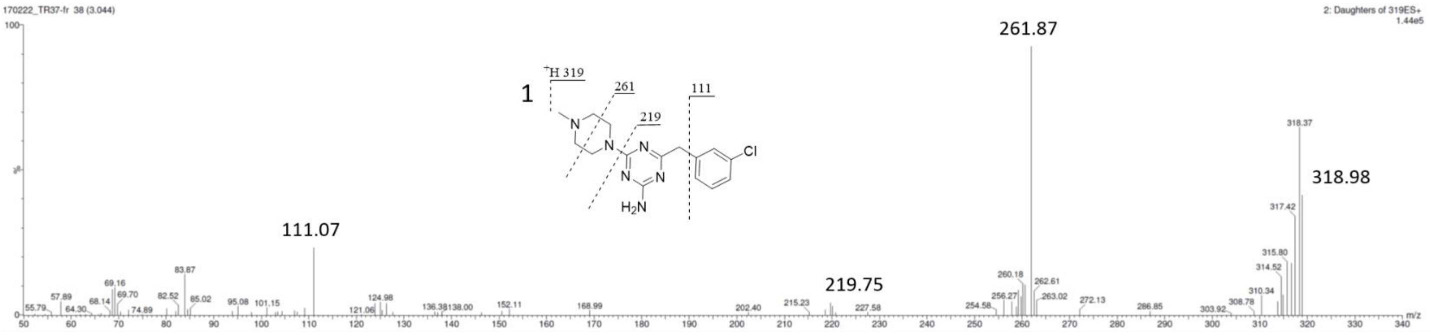

Figure S1. MS spectrum of compound **1** (A) and its MS ion fragment analysis (B).

A

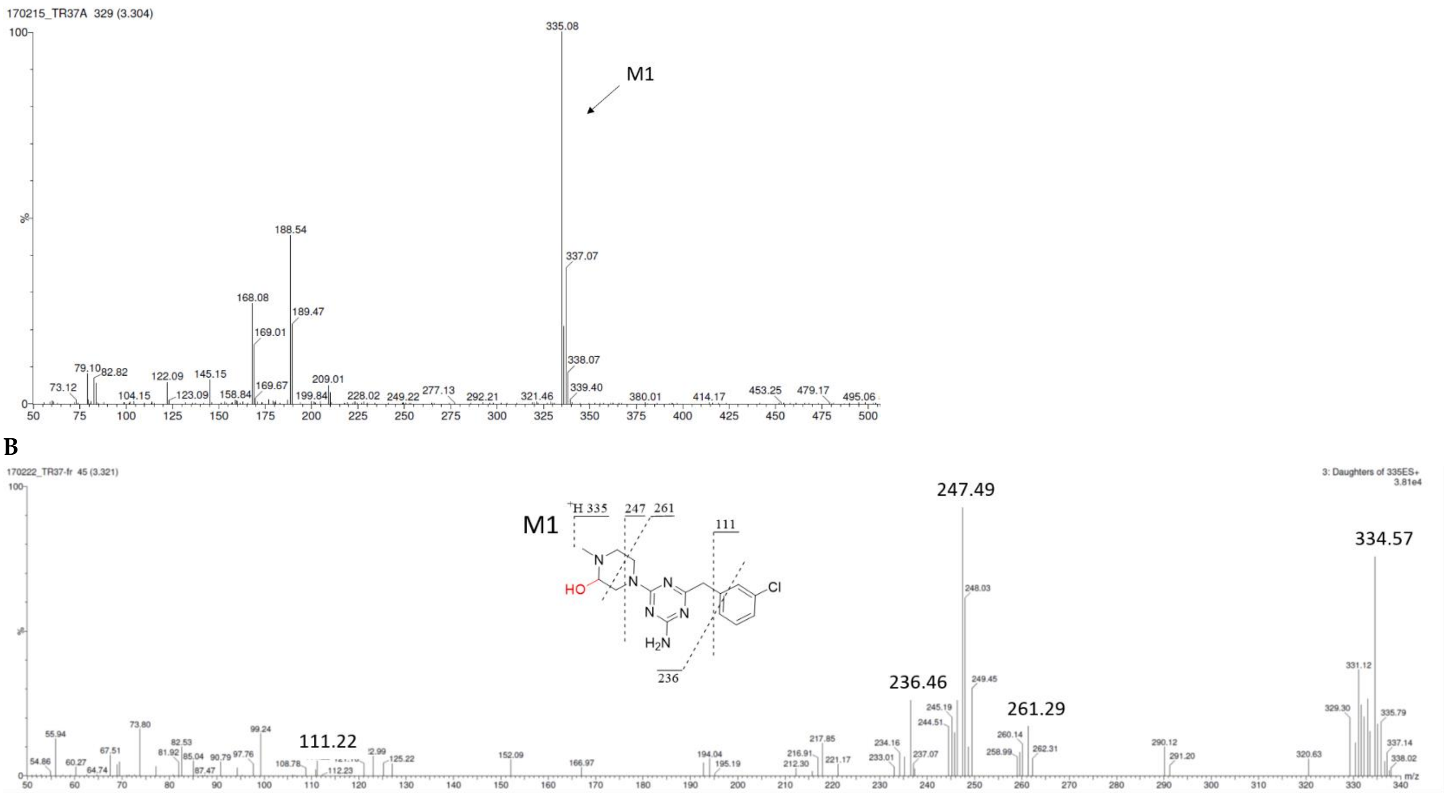

Figure S2. MS spectrum of compound's **1** metabolite M1 (A) and its MS ion fragment analysis (B).

A

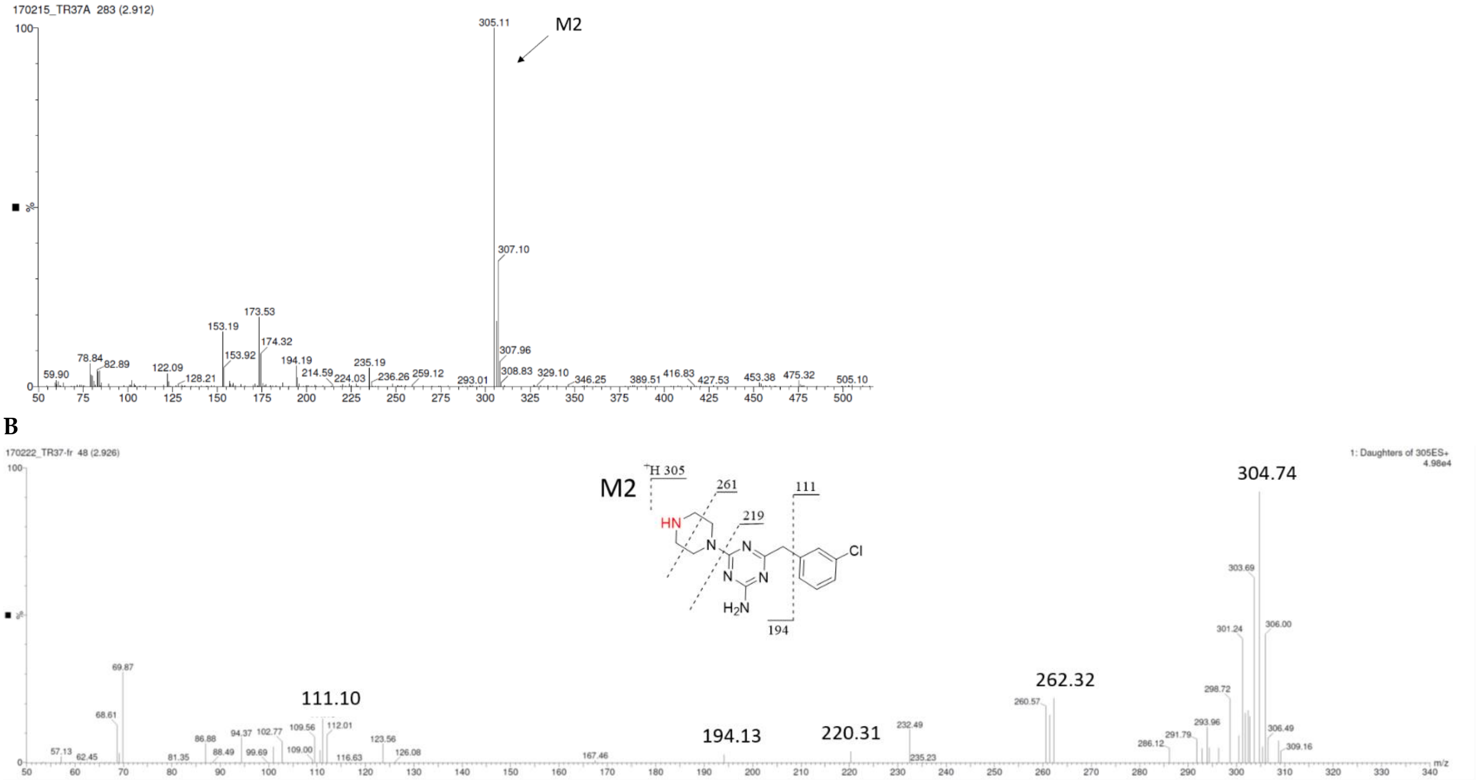

Figure S3. MS spectrum of compound's **1** metabolite M2 (A) and its MS ion fragment analysis (B).

2. MS spectra and MS ion fragment analyses of compound 2 and its metabolites.

A

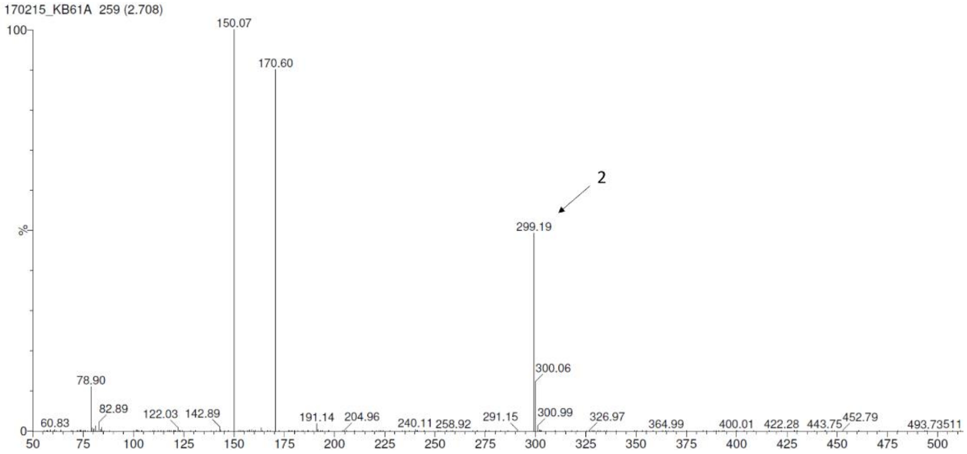

B

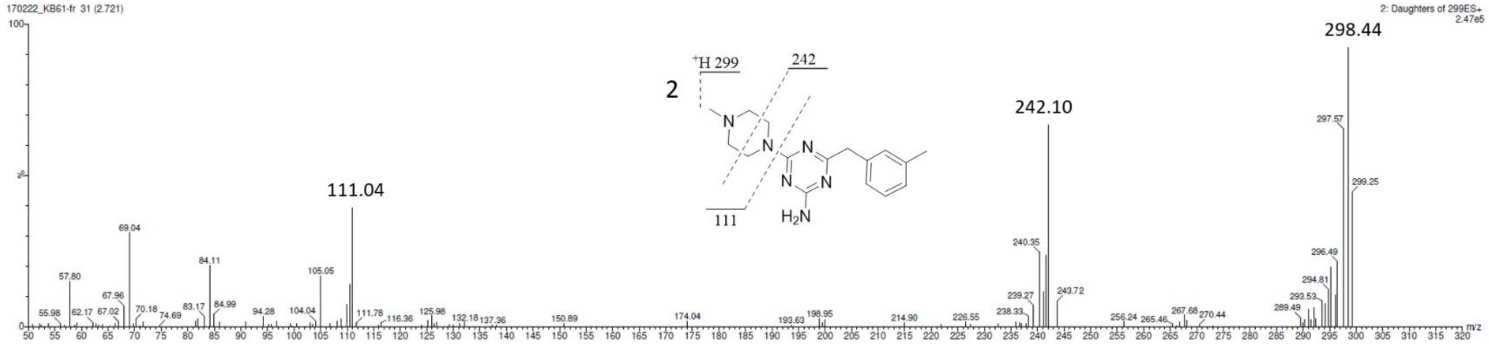

Figure S4. MS spectrum of compound **2** (A) and its MS ion fragment analysis (B).

A

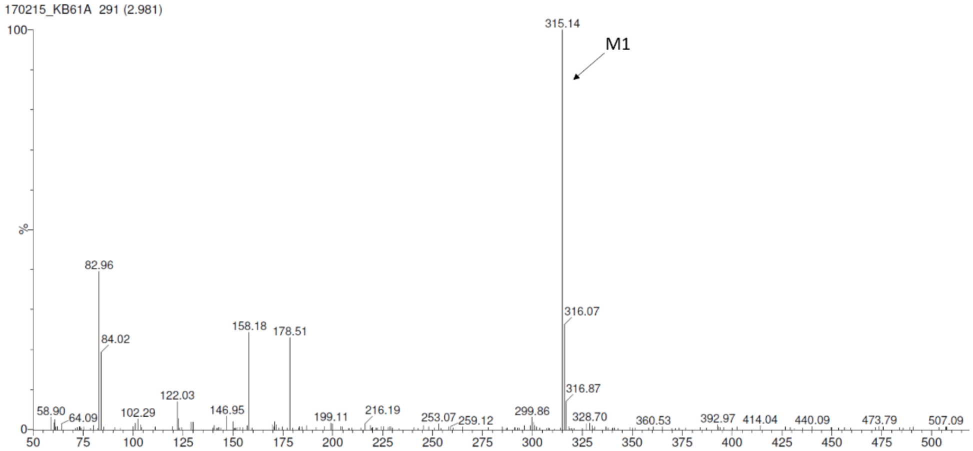

B

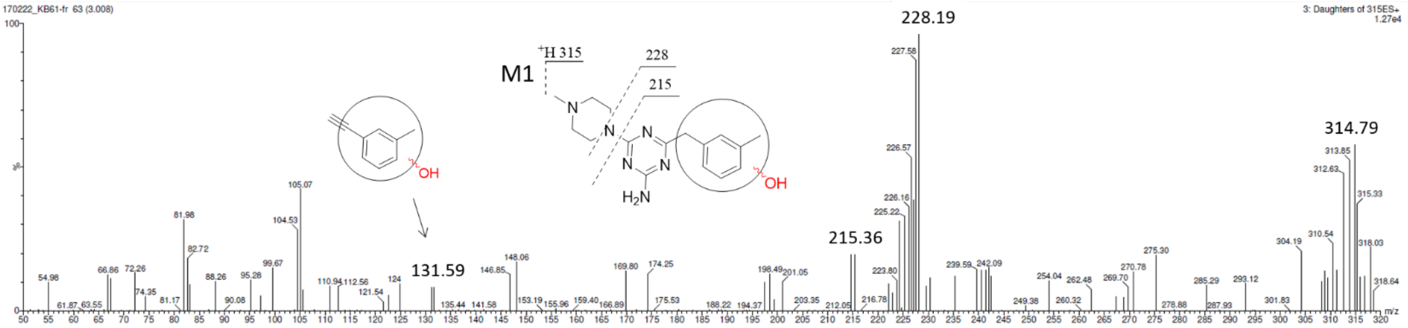

Figure S5. MS spectrum of compound’s **2** metabolite M1 (A) and its MS ion fragment analysis (B).

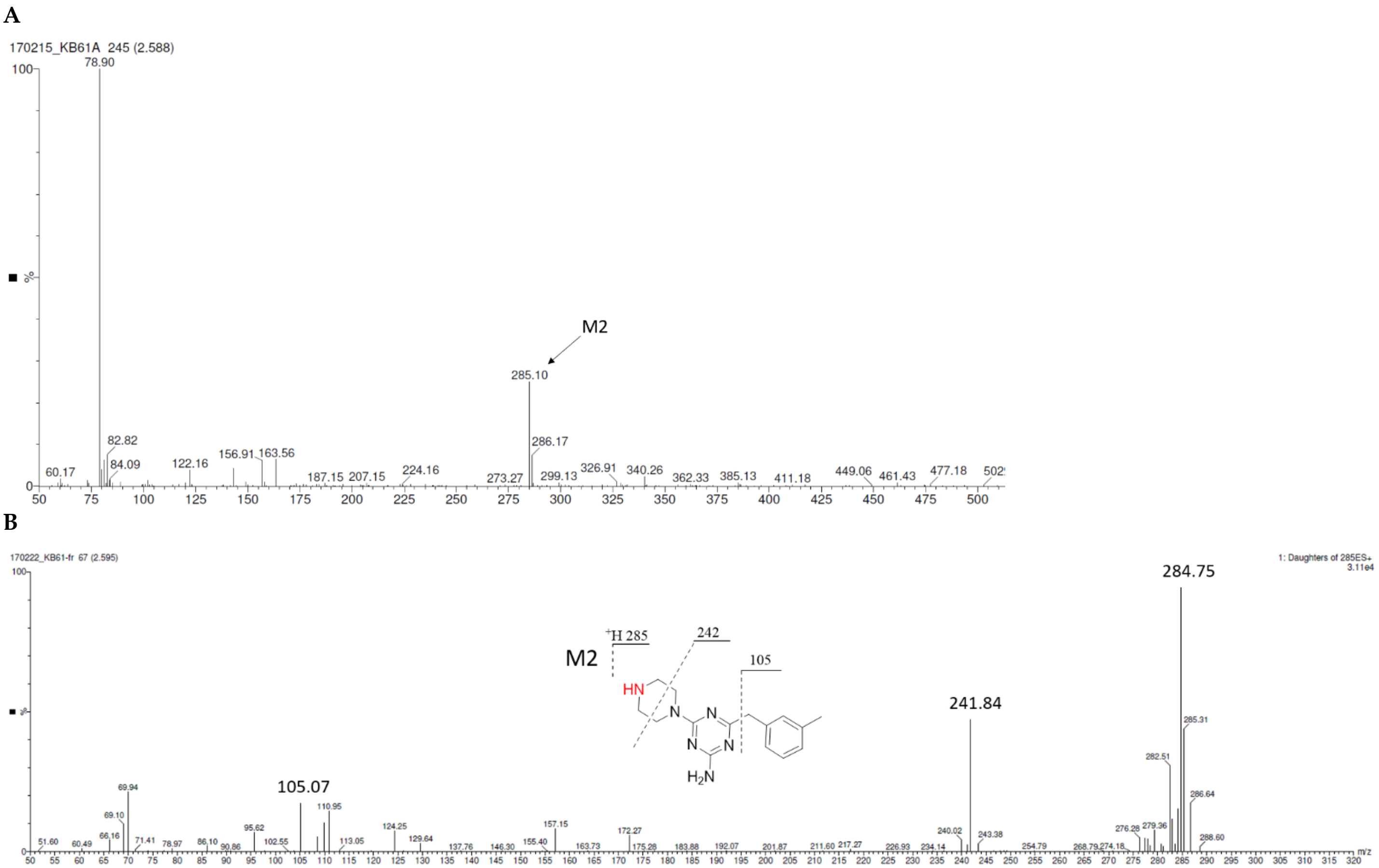

Figure S6. MS spectrum of compound's **2** metabolite M2 (A) and its MS ion fragment analysis (B)

3. MS spectra and MS ion fragment analyses of compound 3 and its metabolites.

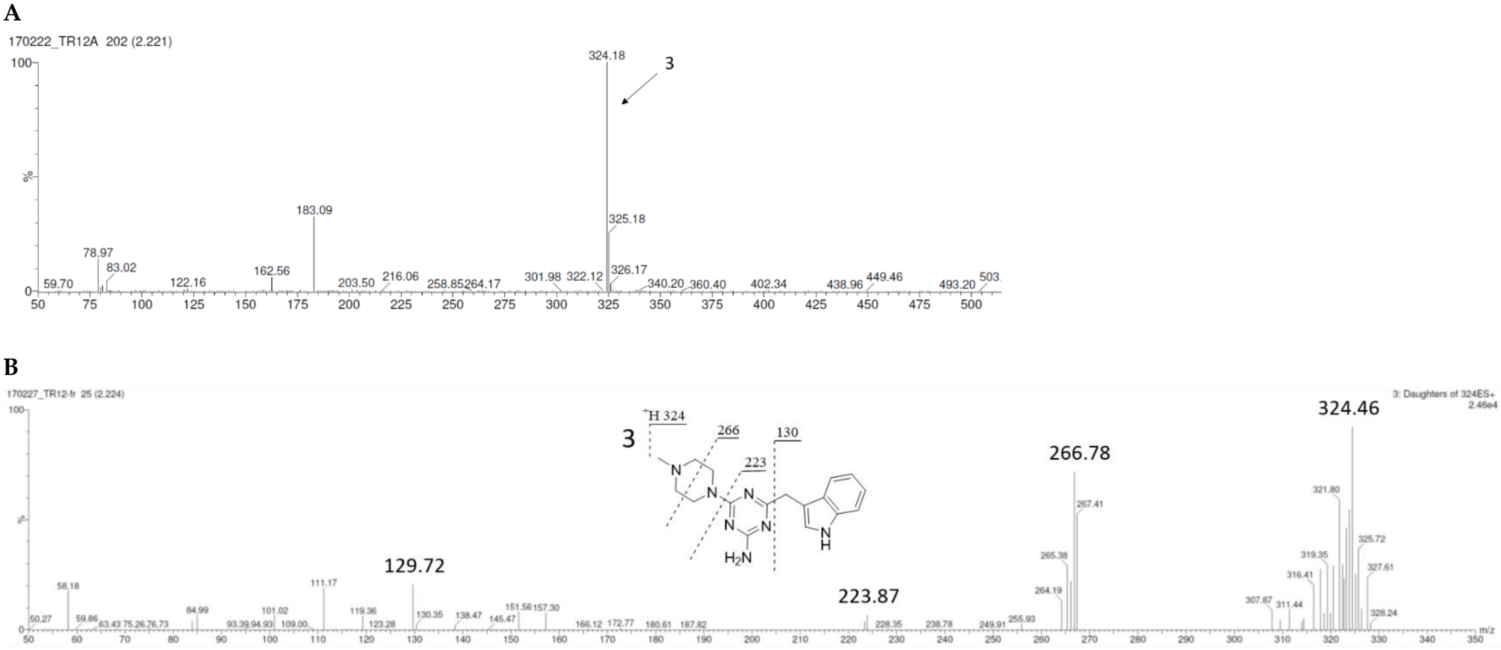

Figure S7. MS spectrum of compound **3** (A) and its MS ion fragment analysis (B).

A

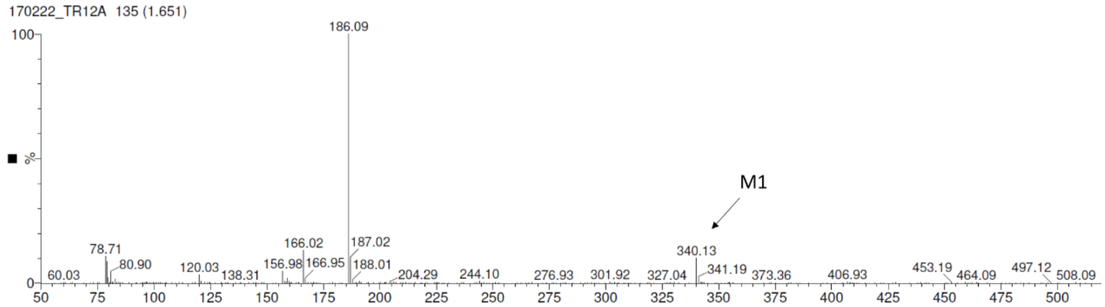

B

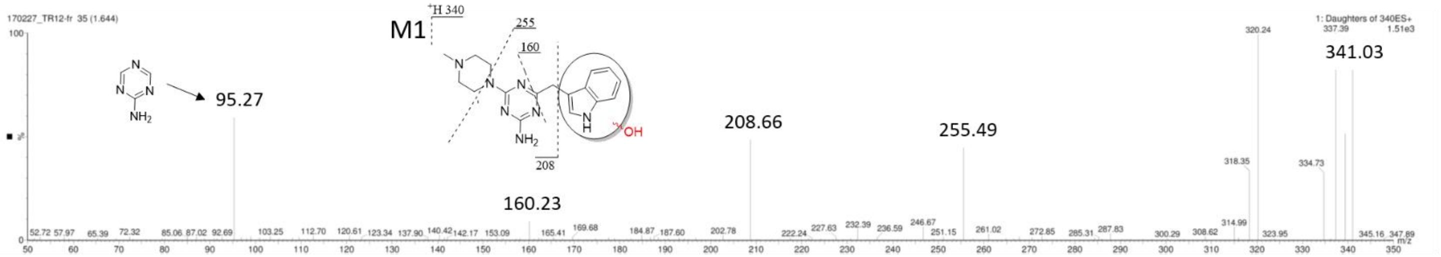

Figure S8. MS spectrum of compound's **3** metabolite M1 (A) and its MS ion fragment analysis (B).

A

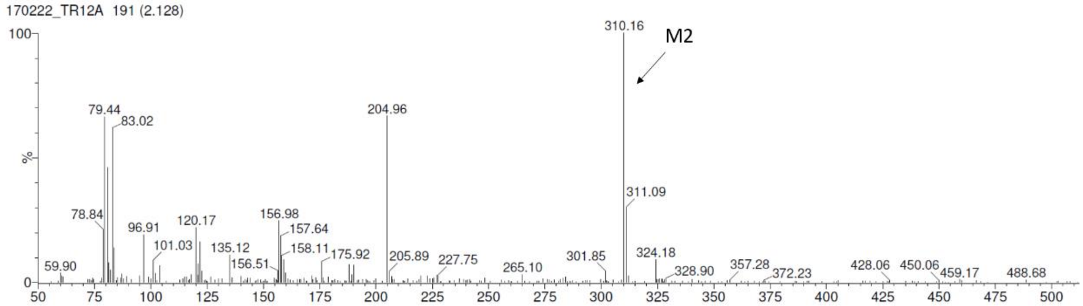

B

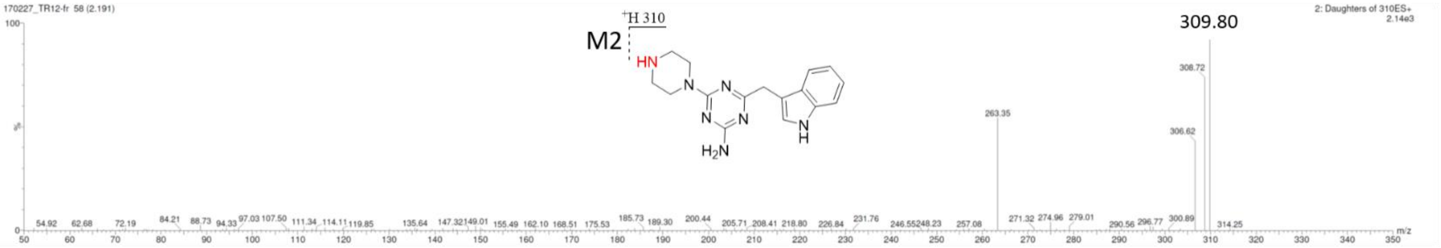

Figure S9 MS spectrum of compound's **3** metabolite M2 (A) and its MS ion fragment analysis (B).

A

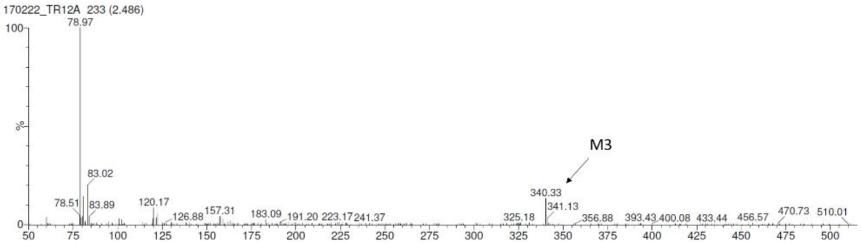

B

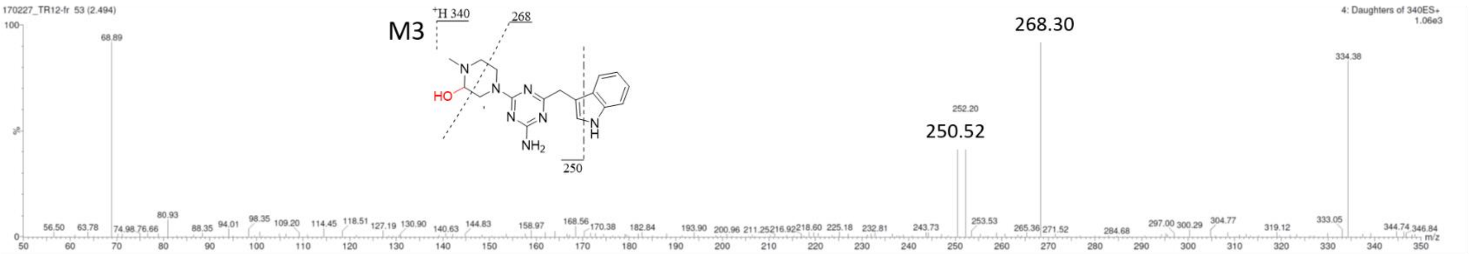

Figure S10 MS spectrum of compound's **3** metabolite M3 (A) and its MS ion fragment analysis (B).

4. MS spectra and MS ion fragment analyses of compound 4 and its metabolites.

A

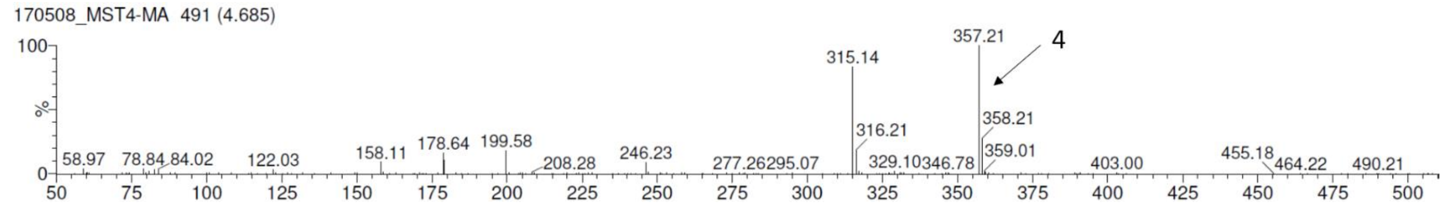

B

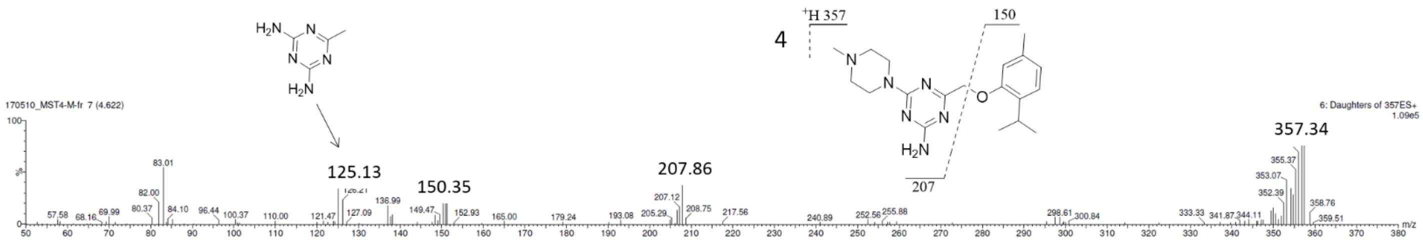

Figure S11. MS spectrum of compound **4** (A) and its MS ion fragment analysis (B).

A

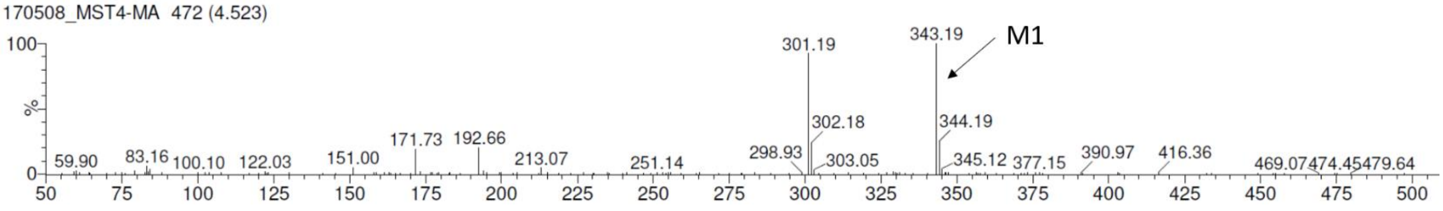

B

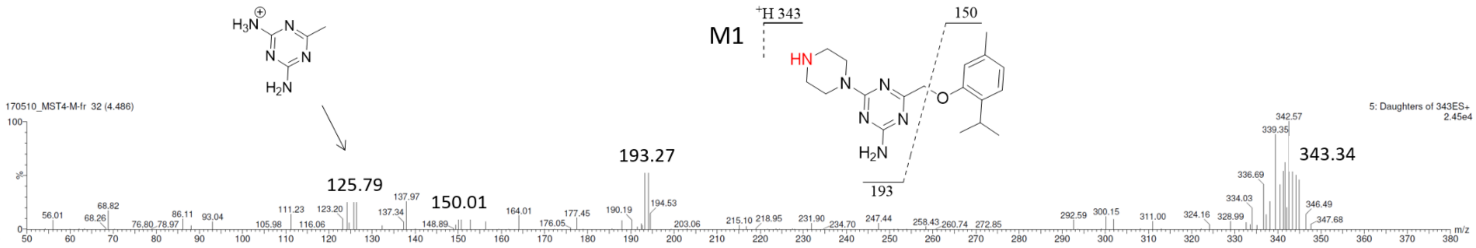

Figure S12 MS spectrum of compound's **4** metabolite M1 (A) and its MS ion fragment analysis (B).

A

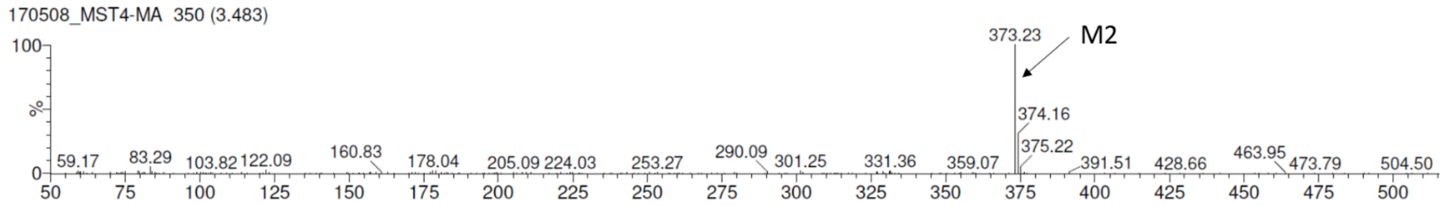

B

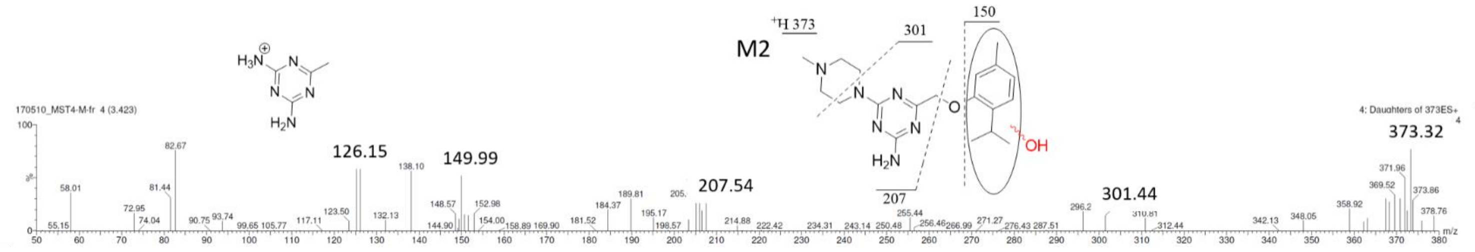

Figure S13 MS spectrum of compound's **4** metabolite M2 (A) and its MS ion fragment analysis (B).

A

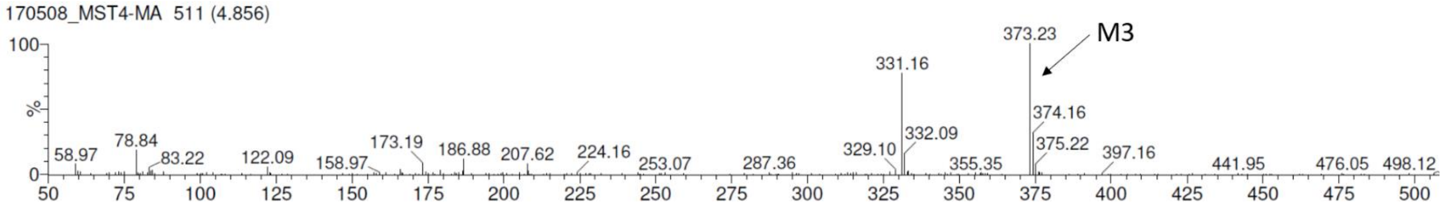

B

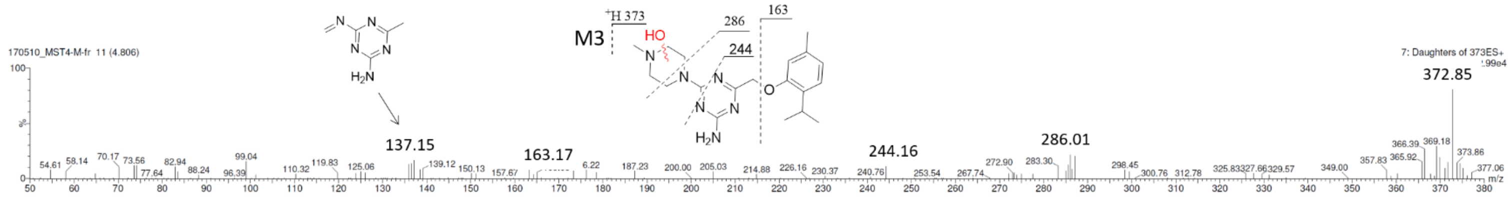

Figure S14 MS spectrum of compound's **4** metabolite M3 (A) and its MS ion fragment analysis (B).

A

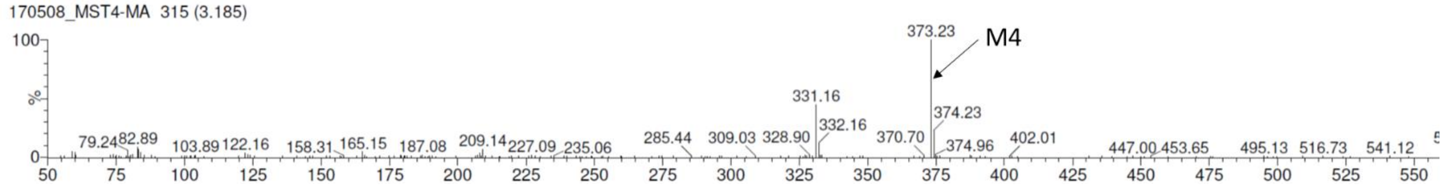

B

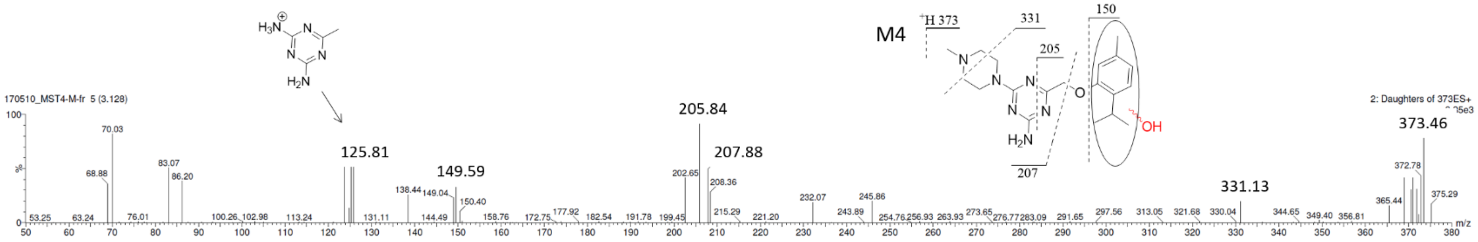

Figure S15 MS spectrum of compound's **4** metabolite M4 (A) and its MS ion fragment analysis (B).

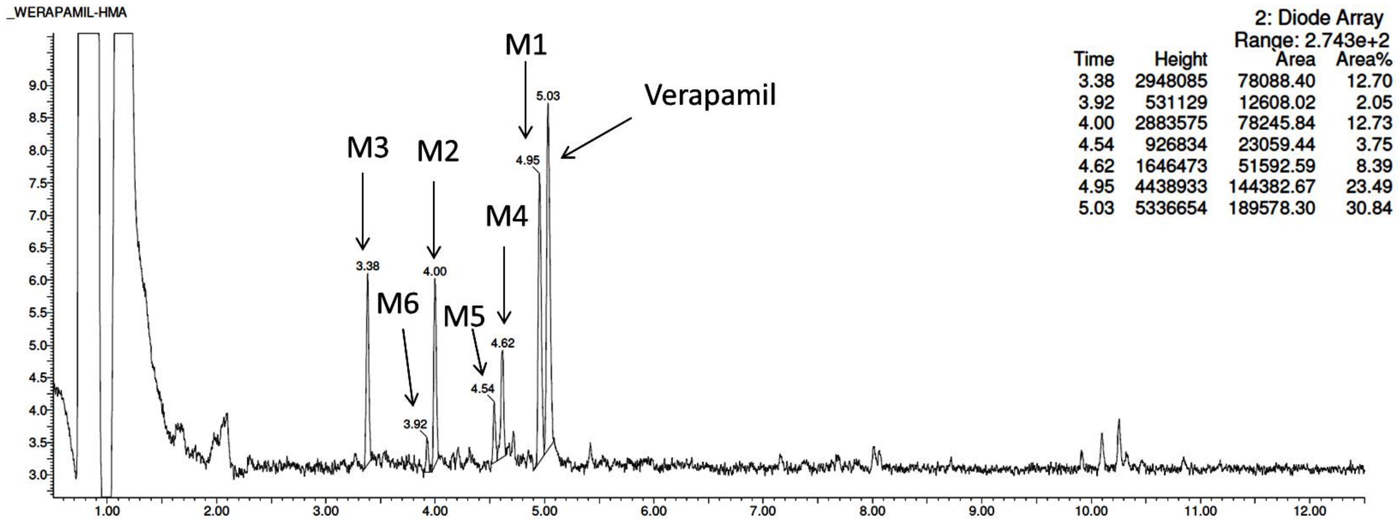

Figure S16 UPLC spectra of the reaction mixture after 120 min incubation of reference drug verapamil with HLMs.
